# Alpha-herpesvirus UL55 synergizes with ICP27 to suppress type I interferon production through conserved and host-adapted mechanisms

**DOI:** 10.1128/jvi.00653-26

**Published:** 2026-06-30

**Authors:** Ying Wu, Mengya Zhang, Anyang Sun, Qiqi Yang, Ruofei An, Mingshu Wang, Shun Chen, Renyong Jia, Mafeng Liu, Qiao Yang, Bin Tian, Xumin Ou, Juan Huang, Di Sun, Dekang Zhu, Xinxin Zhao, Shaqiu Zhang, Yu He, Zhen Wu, Anchun Cheng

**Affiliations:** 1 Engineering Research Center of Southwest Animal Disease Prevention and Control Technology, Ministry of Education of the People’s Republic of China, Chengdu, China; 2 International Joint Research Center for Animal Disease Prevention and Control of Sichuan Province, Chengdu, China; 3 Agricultural Animal Diseases and Veterinary Public Health Key Laboratory of Sichuan Province, Chengdu, China; 4 Avian Disease Research Center, College of Veterinary Medicine of Sichuan Agricultural University, Wenjiang, China; 5 Institute of Veterinary Medicine and Immunology Drugs, Veterinary Department in College of Animal Science, State Key Laboratory of Green Pesticide, GuiZhou University, GuiYang, People's Republic of China; University of Virginia, Charlottesville, Virginia, USA

**Keywords:** duck plague virus (DPV), immune evasion, IFN-I suppression, ICP27-UL55 axis, alpha-herpesvirus, host-adapted evolution

## Abstract

Herpesviruses employ sophisticated immune evasion strategies to establish lifelong infections, subverting type I interferon (IFN-I) responses critical for antiviral defense. However, their adaptive mechanisms across species remain poorly characterized. Using duck plague virus (DPV)—an avian alphaherpesvirus model—we identify a cooperative immune evasion axis wherein ICP27 orchestrates UL55-mediated immunosuppression through dual regulatory mechanisms: its RNA-binding domain (RGG) facilitates UL55 mRNA nuclear export, while its C-terminal domain (CTD) stabilizes UL55 protein via direct interaction. This partnership enables synergistic suppression of IFN-I signaling—co-expression of ICP27 and UL55 inhibits Poly(I:C)-induced immune genes (IFN-β, Mx, OASL, IL-6) more potently than either protein alone. UL55 functions as a precision-targeted IFN-I antagonist, selectively degrading RIG-I and IRF7 through proteasomal pathways—confirmed by proteasome inhibitor rescue (MG132), structural modeling (AlphaFold), and binding assays (Co-IP). Evolutionarily, UL55 homologs (DPV, Herpes simplex virus type 1 [HSV-1], Varicella zoster virus [VZV]) conserve RIG-I targeting but diverge in IRF3/IRF7 regulation—adaptations shaped by UL55 sequence divergence (38.68% identity) and host biology (e.g., waterfowl IRF3 deficiency). This work establishes ICP27-UL55 as a key regulatory axis in herpesviral immune evasion and redefines UL55 as a conserved yet adaptable immunosuppressor in *Alphaherpesvirinae*.

**IMPORTANCE:**

This study fundamentally advances herpesvirology by defining a novel immune evasion paradigm in duck plague virus. We reveal ICP27 as a master regulator that coordinates UL55 immunosuppression through a two-tiered mechanism: RGG domain-mediated mRNA nuclear export and CTD-dependent protein stabilization—an unreported strategy in herpesviruses. UL55 selectively degrades RIG-I and IRF7 via proteasomal pathways, enabling precise IFN-I suppression with minimal immune activation. Crucially, ICP27-UL55 synergy inhibits Poly(I:C)-induced immune genes (IFN-β, Mx, OASL, IL-6) more effectively than individual proteins. Evolutionary analyses demonstrate conserved targeting of RIG-I across alphaherpesvirus UL55 homologs (DPV, HSV-1, VZV) but host-adapted divergence in IRF3/IRF7 regulation, shaped by UL55 sequence variation (38.68% identity) and host biology (e.g., avian IRF3 deficiency). These findings provide the first evidence of effector coordination through integrated transcriptional/post-translational regulation in herpesviruses. Disrupting ICP27-UL55 interaction offers new antiviral targets, while UL55-deficient strains serve as vaccine candidates for poultry disease control.

## INTRODUCTION

Herpesviruses have evolved intricate mechanisms to evade host innate immune responses, with type I interferon (IFN-I) evasion playing a pivotal role in their pathogenesis and persistence (1–5). In the type I interferon (IFN-I) system, the cGAS-STING (6) and RIG-I/MDA5(7) pathways serve as sentinels for viral nucleic acids, activating IRF3/IRF7-dependent transcription of antiviral genes, including IFN-β. While cGAS-STING is classically associated with DNA sensing, emerging evidence highlights the pivotal role of RIG-I-like receptors (RLRs) in countering herpesviral infections through RNA surveillance—even in DNA viruses where viral or host-derived immunostimulatory RNAs may activate this pathway (8–10). While herpesviruses typically encode their immunosuppressive proteins (11–14) to target the core nodes (e.g., PRR/STING/IRFs/STATs) of the IFN pathways through protein-protein interactions (15, 16) or modulation of host gene expression (13, 14), substantial heterogeneity exists in their molecular implementations—from virus-specific effector proteins (e.g., HSV-1 ICP0 vs PRV UL13) to host-pathogen co-adaptation mechanisms (17–19).

Within this context, non-mammalian alphaherpesviruses represent an underexplored frontier. Duck plague virus (DPV), an avian alphaherpesvirus causing lethal outbreaks in waterfowl (20), exemplifies this divergence. Unlike mammalian alphaherpesviruses, DPV efficiently suppresses avian IRF7-mediated IFN responses despite lacking homologs of canonical immune evasion effectors (e.g., ICP0, ICP34.5) (21–23). This distinctive phenotype suggests DPV has evolved unique molecular strategies to disable avian antiviral defenses, yet the identity and mechanisms of key effectors remain unresolved.

Our preliminary work identified ICP27 as a dual-function immune modulator in DPV. As a conserved regulator of viral RNA processing (24, 25) and nuclear export (26, 27), ICP27 exhibits intrinsic IFN-I suppression activity (*unpublished data*) alongside potential effector coordination roles. To systematically map its immune evasion networks, we leveraged our established DPV reverse genetics system (28) to engineer *DPV*-ICP27-FLAG—a recombinant virus expressing C-terminally FLAG-tagged ICP27 with unaltered replication kinetics (Fig. S1A and B). Immunoprecipitation of ICP27-FLAG complexes coupled with LC-MS/MS proteomics identified multiple viral and host interactors (Fig. S1C). Among these, UL55—a protein of unknown function—was detected with statistical significance (Fig. S1D). Although its spectral count was moderate, UL55’s conservation across alphaherpesviruses and DPV literature contained speculative annotations of UL55 homologs in immune evasion (23) warranted further investigation. We therefore hypothesized that UL55 is a DPV-specific immune evasion effector functionally coordinated by ICP27.

Here, through integrated mechanistic and cross-species analyses, we demonstrate that ICP27 orchestrates UL55 expression through a dual-regulatory mechanism: its RGG domain binds UL55 mRNA to enhance nuclear export, while its CTD stabilizes UL55 protein via direct interaction; UL55 functions as a multi-target IFN-I antagonist, selectively degrading RIG-I and IRF7 through interaction and proteasomal pathways; ICP27 and UL55 synergistically amplify IFN-I suppression, establishing a cooperative evasion axis essential for DPV pathogenesis; evolutionary analysis reveals conserved targeting of RIG-I by UL55 homologs (DPV, HSV-1, VZV), but divergent regulation of IRF3/IRF7—a host-adapted strategy shaped by phylogenetic distance between avian and mammalian immune systems. Our work reveals how α-herpesviruses balance universal tactics with host-specific adjustments to evade immunity, offering new targets for therapies against these widespread pathogens.

## RESULTS

### ICP27 C-terminal domain mediates specific interaction with the 80–116 AA domain of UL55 protein

To identify viral proteins potentially associated with ICP27 during DPV infection, we performed immunoprecipitation followed by mass spectrometry (IP–MS/MS) using cells infected with ICP27-Flag–tagged virus (Fig. S1A through C). The analysis identified a range of viral and host proteins in the immunoprecipitates (Fig. S1D; Table S1). In this study, we focused on viral proteins, particularly those with potential roles in immune regulation. To evaluate the reliability of the IP–MS/MS data set, several candidate viral and host proteins identified in the screen were selected for independent validation by co-immunoprecipitation. These candidates showed reproducible and expected interactions in follow-up assays, such as co-immunoprecipitation (Fig. S2), supporting the overall quality of the data set.

**Fig 1:**
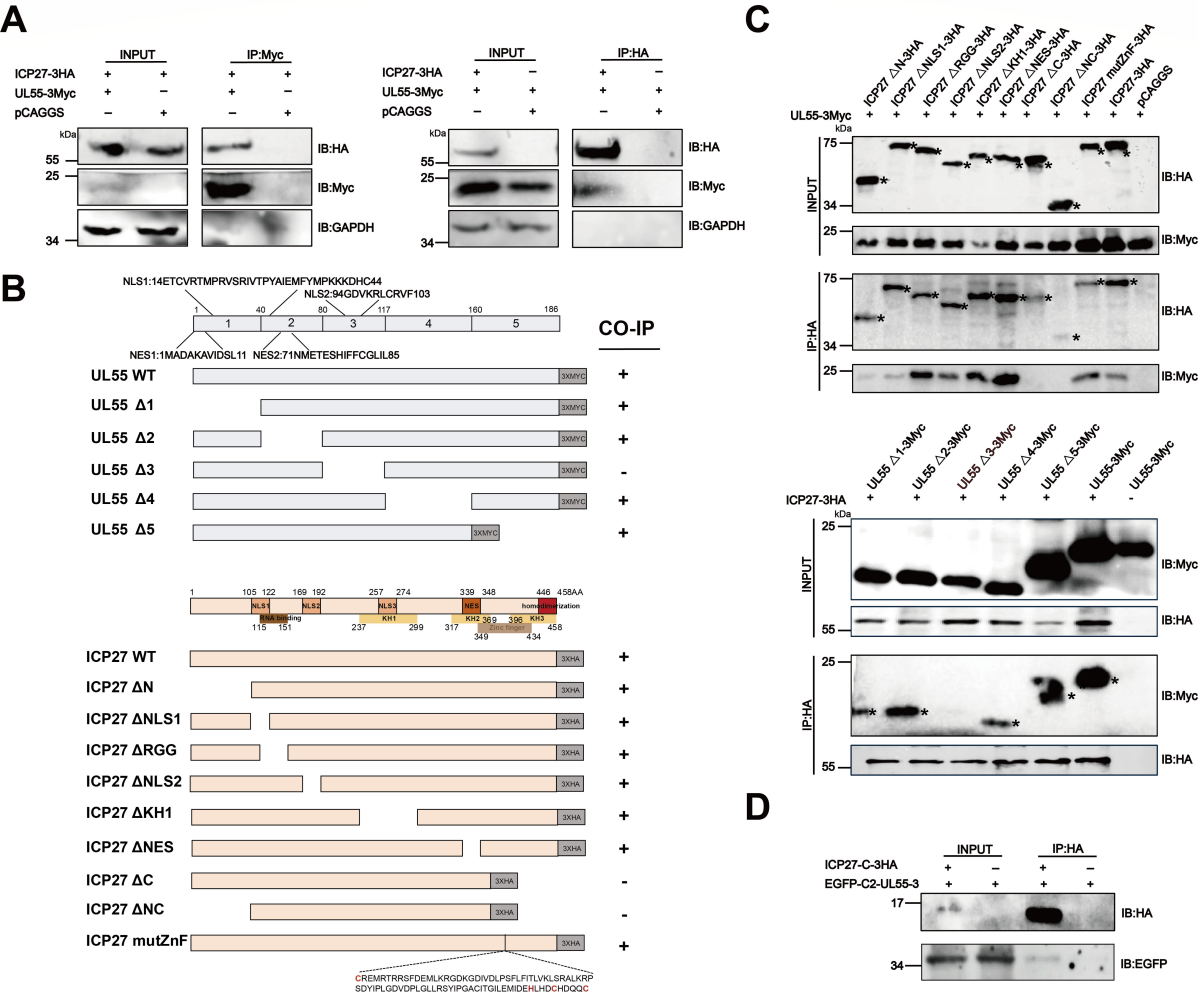
Interaction and mapping of the binding regions between ICP27 and UL55. (**A**) Coimmunoprecipitation (Co-IP) analysis of ICP27-UL55 interaction. Duck embryo fibroblasts (DEFs) were co-transfected with plasmids expressing C-terminally 3HA-tagged ICP27 (pCAGGS-ICP27-3HA) and C-terminally 3Myc-tagged UL55 (pCAGGS-UL55-3Myc), alongside an empty pCAGGS plasmid as a vector control. Cells were harvested 24 h post-transfection. Lysates were subjected to immunoprecipitation with anti-Myc antibody to enrich UL55 complexes or anti-HA antibody to enrich ICP27 complexes. Immunoprecipitates and whole-cell lysates (Input) were analyzed by Western blotting using anti-HA and anti-Myc antibodies to detect the indicated proteins. (**B**) Schematic representation of UL55 and ICP27 truncation mutants. Putative functional domains within ICP27 and UL55 are indicated. Truncation constructs used for mapping are shown below each wild-type protein. The “+” and “–” symbols denote interaction or no interaction, respectively, as determined by subsequent Co-IP assays. (**C**) Mapping interaction domains by reciprocal Co-IP. DEFs co-transfected with truncation mutants of pCAGGS-ICP27-3HA and pCAGGS-UL55-3Myc were harvested 24 h post-transfection. Interactions were assessed by anti-HA or anti-Myc immunoprecipitation, followed by Western blot analysis. Asterisks (*) were added to label the corresponding protein bands of ICP27, UL55, and their truncated proteins. (**D**) Identification of minimal interaction domains. Co-IP using anti-HA antibody followed by Western blotting confirmed specific binding between the C-terminal domain of ICP27 (ICP27-C-3HA) and UL55 amino acids 80–116 (EGFP-C2-UL55-3). DEFs were co-transfected with respective truncation mutants, alongside empty pCAGGS vector controls. Cells were harvested 24 h post-transfection for analysis.

Among the viral candidates, an uncharacterized protein UL55, which has potential relevance to immune regulation, was detected with limited peptide coverage. Given that proteins with low abundance or transient interactions are often underrepresented in MS-based data sets, this result was considered preliminary. Therefore, the IP–MS/MS analysis was used as an initial screening step to nominate candidate proteins for further investigation, rather than as definitive evidence of interaction. To validate UL55 as a functional ICP27 interactor predicted by IP LC-MS screening, we performed co-immunoprecipitation (Co-IP) in duck embryo fibroblasts (DEFs) transfected with full-length ICP27 and UL55 expression constructs. Anti-Myc immunoprecipitation of UL55-3Myc robustly co-precipitated ICP27, confirming their physical association in a heterologous expression system. Reciprocal Co-IP using anti-HA antibody against ICP27-3HA similarly enriched UL55 (Fig. 1A), establishing a bona fide protein-protein interaction. To delineate interaction domains, we constructed truncation mutants based on predicted functional domains (Fig. 1B). Reciprocal Co-IP revealed that the C-terminal domain (CTD) of ICP27 specifically binds residues 80-116 of UL55 (Fig. 1C). Given that ICP27 CTD mediates oligomerization in homologs (e.g., HSV-1 ICP27) and it has the possibility that the interaction requires N-terminal sequences in the context of a dimer or some higher oligomer (1, 29). To exclude this claim, minimal binding domain analysis were performed and demonstrated that ICP27 CTD alone suffices to precipitate the UL55 (80–116) fragment, confirming only CTD-dependent recognition (Fig. 1D).

### ICP27 modulates UL55 expression through dual mechanisms

**Fig 2:**
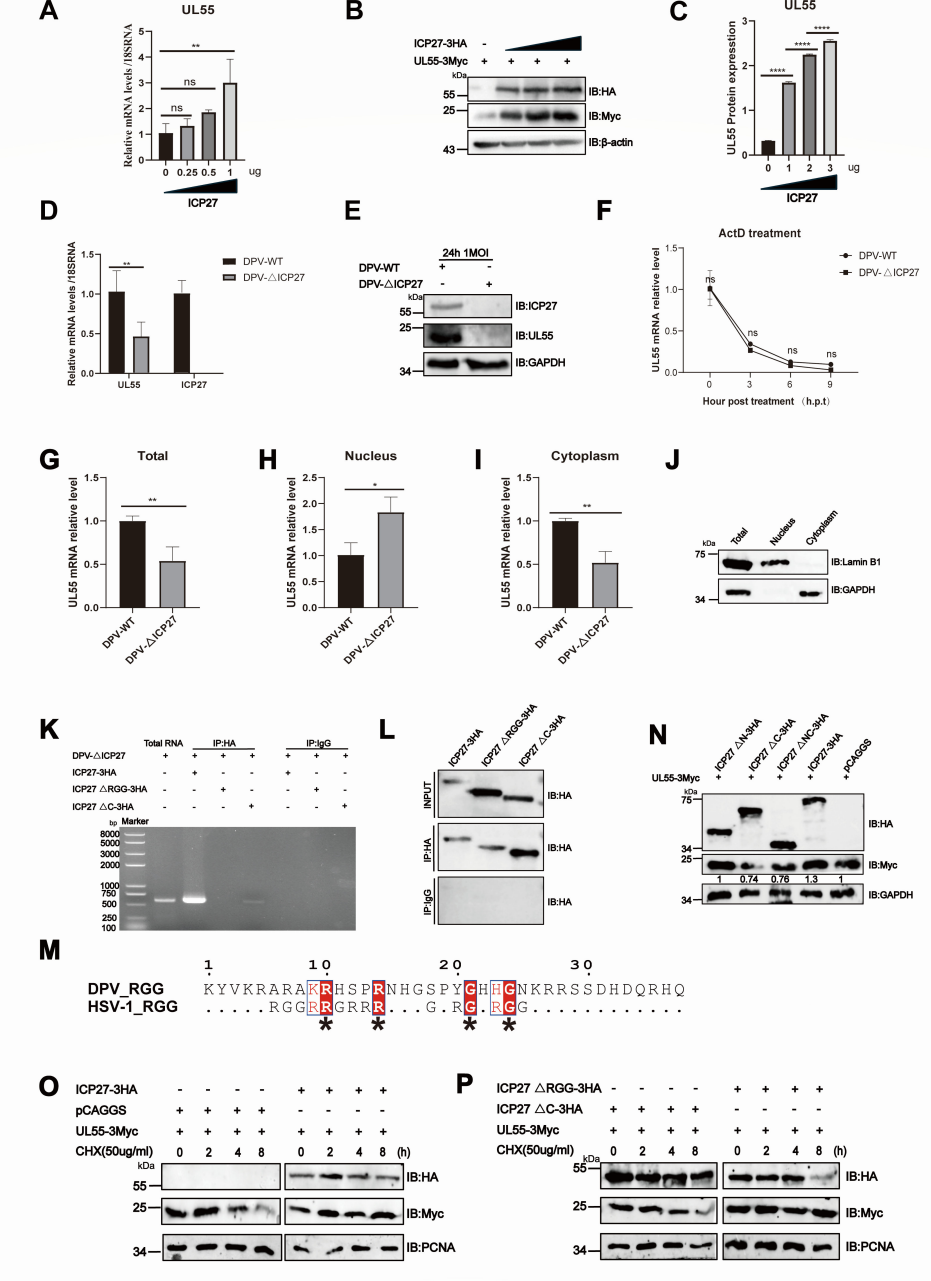
ICP27 post-transcriptionally and post-translationally upregulates UL55. (**A–C**) ICP27 enhances UL55 expression dose-dependently. DEFs were co-transfected with pCAGGS-UL55-3Myc and increasing amounts of pCAGGS-ICP27-3HA. Cells were harvested at 24 h post-transfection. UL55 mRNA (**A**) and protein (**B**) levels were measured by RT-qPCR and Western blot. UL55 protein expression in (**B**) was quantified by grayscale analysis using ImageJ (**C**). Results of the above data were compared using one-way ANOVA. Significance is indicated as follows: ns, not significant; *P < 0.05; **P < 0.01; ***P < 0.001; ****P < 0.0001. (**D and E**) ICP27 deletion reduces UL55 mRNA and protein levels. DEFs were infected with DPV-ΔICP27 or DPV-WT (MOI = 1). Cells were harvested at 24 h post-infection (hpi). UL55 mRNA (**D**) and protein (**E**) levels were determined by RT-qPCR and Western blot, respectively. Results of the above data were compared using one-way ANOVA. Significance is indicated as follows: ns, not significant; *P < 0.05; **P < 0.01; ***P < 0.001; ****P < 0.0001. (**F**) ICP27 minimally affects UL55 mRNA stability. DEFs infected with DPV-ΔICP27 or DPV-WT (MOI = 1) were treated with 2 μg/mL actinomycin D (Act D) at 24 hpi. Cells were harvested at 0, 3, 6, and 9 h post-treatment. UL55 mRNA levels were analyzed by RT-qPCR. Results of the above data were compared using one-way ANOVA. Significance is indicated as follows: ns, not significant; *P < 0.05; **P < 0.01; ***P < 0.001; ****P < 0.0001. (**G–J**) ICP27 promotes UL55 mRNA nuclear export. DEFs infected with DPV-ΔICP27 or DPV-WT (MOI = 1) were harvested at 12 hpi for nuclear-cytoplasmic fractionation. UL55 mRNA levels in total lysates (**G**), nuclear (**H**), and cytoplasmic (**I**) fractions were measured by RT-qPCR and normalized by 18sRNA. Fractionation efficiency was validated by Western blot for LaminB1 (nuclear marker), and GAPDH (cytoplasmic marker) (**J**). The results were compared using the *t*-test, and the significance analysis is as follows: ns indicates no significant difference; *P* < 0.05; *P* < 0.01; *P* < 0.001; *P* < 0.0001. (**K**) ICP27 binds UL55 mRNA via its RGG domain. RIP assays assessed UL55 mRNA binding in DEFs infected with ICP27-deficient virus (MOI = 1) and transfected with pCAGGS-ICP27-3HA, pCAGGS-ICP27△RGG-3HA, or pCAGGS-ICP27△C-3HA for complementation. Cells were harvested at 12 hpi. (**L**) Expression and RIP validation of ICP27 mutants. Enrichment of ICP27, ICP27 △RGG, and ICP27 △C proteins in panel F samples was confirmed by Co-IP assays. (**M**) Conservation of the RGG motif. Sequence alignment of HSV-1 ICP27-RGG and DPV ICP27-RGG domains. Asterisks (*) denote conserved RGG motifs. (**N**) The CTD of ICP27 is essential for UL55 upregulation. DEFs co-transfected with pCAGGS-UL55-3Myc and pCAGGS-ICP27-3HA, pCAGGS-ICP27△N-3HA, pCAGGS-ICP27△C-3HA, pCAGGS-ICP27△NC-3HA, or empty pCAGGS vector were harvested at 24 h post-transfection. UL55 protein levels were detected by Western blot. (**O and P**) ICP27 stabilizes UL55 protein via CTD-mediated interaction. DEFs co-transfected with pCAGGS-UL55-3Myc and wild-type ICP27 (**O**) or truncated ICP27 plasmids (**P**) were treated with 50 μg/mL cycloheximide (CHX) at 24 h post-transfection. Cells were harvested at 0, 2, 4, and 8 h post-CHX treatment. UL55 protein levels were analyzed by Western blot.

To elucidate the functional consequences of ICP27-UL55 interaction, we first assessed ICP27’s impact on UL55 expression. As shown in Fig. 2A through C, ICP27 overexpression in transfected DEFs dose-dependently enhanced UL55 expression—mRNA increased 3-fold and protein levels rose proportionally at maximal ICP27 concentration. Critically, this regulation was ICP27-specific: ICP22 (a control viral protein) failed to alter UL55 expression (Fig. S3A and B), confirming targeted regulatory activity. Further, we examined both the mRNA and protein levels of UL55 following DPV infection. As shown in Fig. 2D, relative quantification confirmed that ICP27 mRNA was readily detected in DPV-WT-infected cells, but was absent in DPV-ΔICP27-infected cells, with expression levels indistinguishable from background, thereby validating the successful gene deletion; while the transcript level of UL55 was reduced by approximately 50% in cells infected with the ICP27-null virus (DPV-ΔICP27) compared with the wild-type virus (DPV-WT). Strikingly, however, UL55 protein was nearly undetectable under DPV-ΔICP27 infection (Fig. 2E). This significant discrepancy between mRNA and protein levels suggested that ICP27’s regulation of UL55 likely extends beyond the transcriptional level. Given the multiple regulatory roles of the ICP27 homologs in processes, such as RNA binding and mRNA processing, we hypothesized that DPV ICP27 might also play a critical role at post-transcriptional (e.g., mRNA export or translation efficiency) or post-translational (e.g., protein stability) levels. This observation prompted our subsequent investigations to validate this hypothesis.

To investigate this mRNA-protein discrepancy of UL55, we next examined whether ICP27 stabilizes UL55 mRNA using actinomycin D (Act D) chase assays (30)—a standard method for quantifying mRNA decay kinetics by inhibiting transcription (31, 32). As shown in Fig. 2F, the UL55 mRNA decay rates were indistinguishable between ΔICP27 and wild-type infections after ActD treatment (Fig. 2F), ruling out mRNA stability effects. We therefore hypothesized that ICP27 regulates nuclear-cytoplasmic trafficking. After confirming the successful separation of nuclear and cytoplasmic fractions by Western blot (Fig. 2J), quantitative PCR (qPCR) was performed to detect UL55 mRNA levels in different cellular components. The results demonstrated that ICP27 deficiency led to abnormal accumulation of UL55 mRNA in the nucleus (significantly higher than that in the wild-type strain), while its cytoplasmic levels showed a marked decrease (Fig. 2G through I). This nuclear trapping phenotype implicated ICP27 in mRNA export facilitation. To validate whether DPV ICP27 binds to UL55 mRNA through its RNA-binding domain (RGG) and promotes its nuclear export, thereby enhancing its translation efficiency, we used the ΔRGG mutant as a functional control to assess the requirement of the RNA-binding domain for ICP27 interaction with UL55 mRNA. The RGG motif is a well-characterized RNA-binding domain containing conserved Arg–Gly–Gly sequence in herpesvirus ICP27 proteins, which is required for interaction with GC-rich RNA sequences. Deletion of this region is therefore expected to abolish the RNA-binding activity of ICP27. As shown in Fig. 2K and L, RNA immunoprecipitation (RIP) confirmed DPV ICP27 bound UL55 mRNA specifically through its RGG domain—ΔRGG mutants lost binding capacity while ΔCTD mutants retained it. Evolutionarily, this function is conserved: sequence alignment showed high identity in conserved amino acids in RGG motifs between DPV and HSV-1 ICP27 (Fig. 2M), consistent with preserved RNA-binding functions across alphaherpesviruses (33–35).

Parallel studies revealed CTD-mediated protein stabilization. Co-expression of UL55 with ICP27 truncation mutants demonstrated that the CTD of ICP27 was the core domain that promoted UL55 expression (UL55 level was not increased in ΔC/ΔNC mutants but in ΔN mutants) (Fig. 2N). We then performed cycloheximide (CHX) chase assays to examine the stability of the UL55 protein affected by ICP27. CHX is a well-known protein synthesis inhibitor used for monitoring the degradation rate of pre-existing proteins (36). The results showed that the degradation rate of UL55 in the co-transfected wild-type ICP27 and UL55 group was significantly lower compared with the control group transfected with vector and UL55 alone, indicating that ICP27 increases the stability of UL55 protein (Fig. 2O). However, deletion of ICP27-CTD but not RGG region abolished this stabilization (Fig. 2P), suggesting that the interaction is very important for UL55 protein expression and stability. Thus, ICP27 coordinates UL55 expression through multi-layered regulation mechanisms: RGG-dependent mRNA binding and nuclear export, and CTD-mediated protein stabilization through interaction.

### ICP27 and UL55 synergistically suppress IFN-I signaling

**Fig 3:**
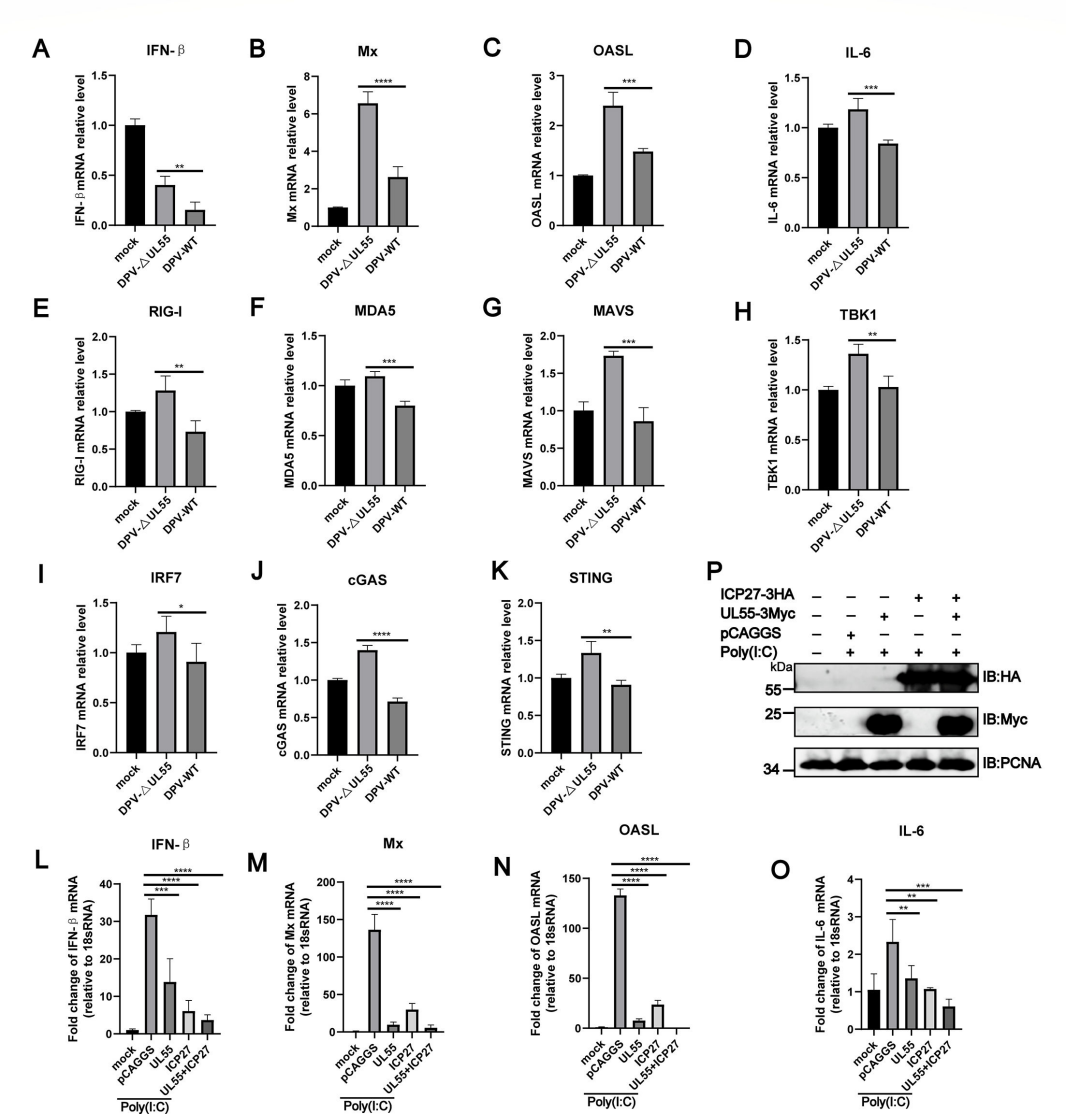
ICP27 and UL55 synergistically promote DPV immune evasion. (**A–K**) UL55 deletion enhances DPV-induced immune gene expression. DEFs infected with DPV-WT or DPV-ΔUL55 (MOI = 0.01) were harvested at 36 hpi. mRNA levels of immune factors (IFN-β, IL-6, Mx, OASL) and IFN-I pathway components (RIG-I, MDA5, MAVS, TBK1, IRF7, cGAS, STING) were quantified by RT-qPCR. Results of the above data were compared using one-way ANOVA. Significance is indicated as follows: ns, not significant; *P < 0.05; **P < 0.01; ***P < 0.001; ****P < 0.0001. (**L–P**) ICP27 and UL55 cooperatively suppress Poly(I:C)-induced immune responses. DEFs were transfected with: pCAGGS (vector control), pCAGGS-UL55-3Myc, pCAGGS-ICP27-3HA, and pCAGGS-UL55-3Myc + pCAGGS-ICP27-3HA (co-transfection). At 24 h post-transfection, cells were mock-treated or transfected with Poly(I:C) for 12 h. Cells were then harvested, and mRNA levels of IFN-β (**L**), Mx (**M**), OASL (**N**), and IL-6 (**O**) were analyzed by RT-qPCR (normalized to 18S rRNA). UL55 protein expression was confirmed by Western blotting (**P**). Results of the above data were compared using one-way ANOVA. Significance is indicated as follows: ns, not significant; *P < 0.05; **P < 0.01; ***P < 0.001; ****P < 0.0001.

While UL55 homologs in alphaherpesviruses remain poorly characterized, preliminary evidence suggested DPV UL55 may modulate the cGAS-STING pathway (23). To determine the role of UL55 in DPV-induced innate immunity, DEF cells were infected with DPV-WT or ΔUL55 virus at a low MOI (0.01) and analyzed at 48 hpi. As shown in Fig. 3A through K, compared with the mock group, DPV-WT infection resulted in a general downregulation of IFN-β and upstream signaling molecules, including RIG-I, MDA5, MAVS, TBK1, IRF7, cGAS, and STING. In contrast, ISG responses were differentially affected: Mx and OASL expression was significantly upregulated (>1.5-fold), whereas IL-6 showed no significant change (<1.5-fold change). This pattern likely reflects the cumulative outcome of multi-round infection and the hierarchical nature of innate immune signaling. At low MOI, infected cells suppress IFN production, while residual or paracrine signaling can still sustain selective ISG expression, particularly for sensitive ISGs such as Mx and OASL. Importantly, deletion of UL55 markedly reversed these effects. Compared with DPV-WT infection, ΔUL55 infection led to significant upregulation of IFN-β, upstream signaling components, and ISGs (Mx, OASL, and IL-6). Although high MOI, single-step infection conditions would better distinguish direct effects from secondary or epistatic effects, the above results are sufficient to demonstrate that UL55 functions as a broad suppressor of innate immune responses during DPV infection. We next tested functional synergy between ICP27 and UL55. As expected, co-expression of ICP27 and UL55 in DEFs suppressed Poly(I:C)-induced IFN-β, Mx, OASL, and IL-6 more potently than either protein alone (Fig. 3L through P)—demonstrating synergistic immune antagonism in DPV. This synergy stems from ICP27’s dual regulation of UL55: By enhancing UL55 mRNA nuclear export and protein stability, ICP27 amplifies UL55’s immunosuppressive capacity, establishing a coordinated evasion axis essential for DPV pathogenesis.

### UL55 is a multi-target inhibitor of type I interferon pathways

**Fig 4:**
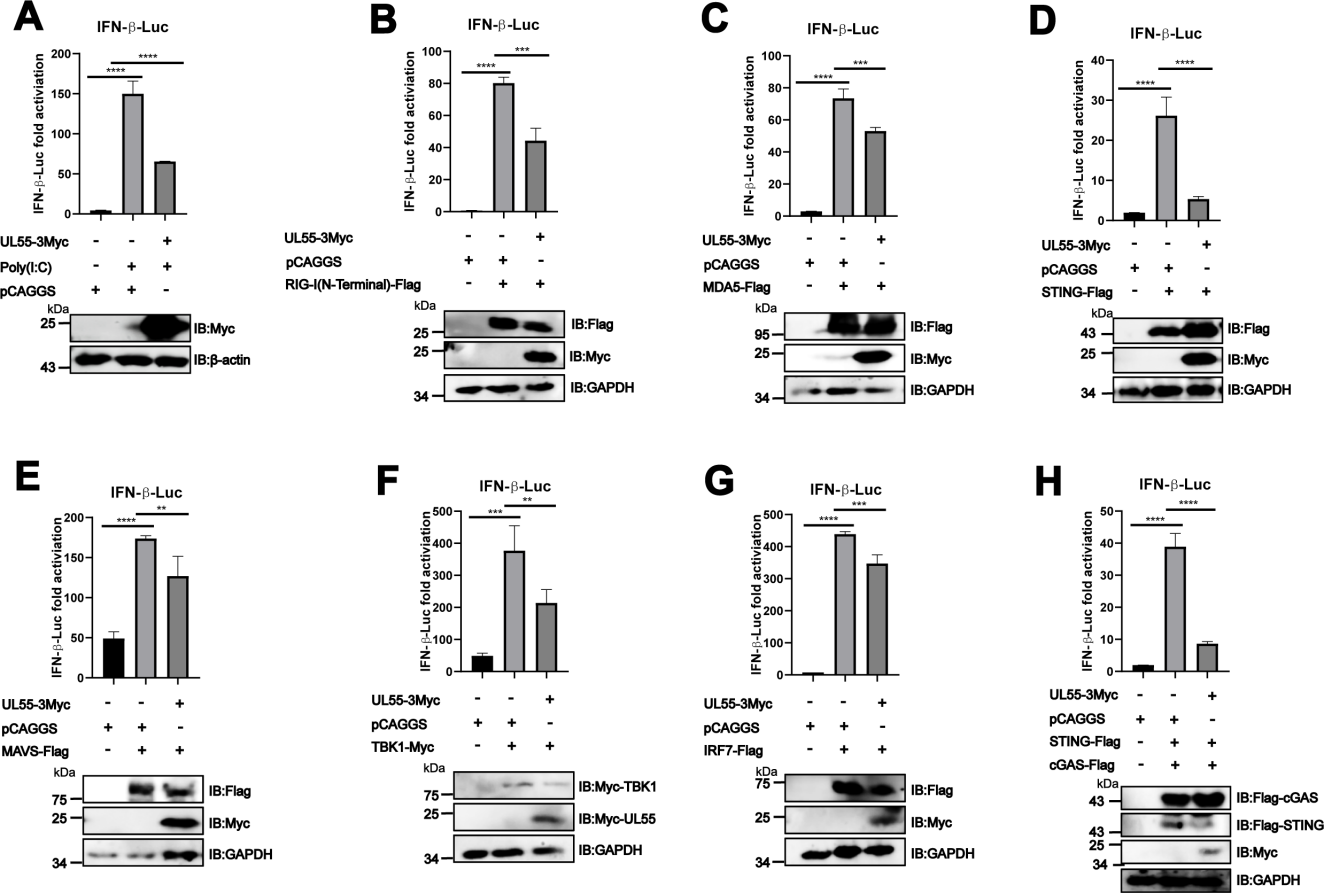
UL55 inhibits DNA/RNA sensing pathway-mediated activation of the IFN-β promoter. (**A**) UL55 suppresses Poly(I:C)-induced IFN-β promoter activity. DEFs were co-transfected with the IFN-β reporter plasmid (IFN-β-Luc), the control plasmid pRL-TK, and either pCAGGS-UL55-3Myc or the empty vector pCAGGS; at 24 h post-transfection, cells were stimulated with Poly(I:C) for 12 h, and IFN-β promoter activity was quantified by dual-luciferase reporter assay. Results of the above data were compared using one-way ANOVA. Significance is indicated as follows: ns, not significant; *P < 0.05; **P < 0.01; ***P < 0.001; ****P < 0.0001. (**B–H**) UL55 inhibits IFN-β promoter activation by cGAS/STING and RIG-I/MDA5 pathway components. For evaluating UL55-mediated suppression of IFN-β promoter activation by key signaling molecules, DEFs were co-transfected with IFN-β-Luc, pRL-TK, either pCAGGS-UL55-3Myc or pCAGGS (vector control), and individual expression plasmids for components of the RIG-I/MDA5 or cGAS/STING pathways: pCAGGS-RIG-I(N-terminal)-Flag, pCAGGS-MDA5-Flag, pCAGGS-MAVS-Flag, pCAGGS-TBK1-Myc, pCAGGS-STING-FLAG, pCAGGS-cGAS-FLAG, or pCAGGS-IRF7-Flag; cells were harvested 36 h post-transfection, and IFN-β promoter activity was measured via dual-luciferase reporter assay. Expression of UL55 and each co-transfected signaling protein was confirmed by Western blot analysis in the lower panels of subfigures A–H. Results of the above data were compared using one-way ANOVA. Significance is indicated as follows: ns, not significant; *P < 0.05; **P < 0.01; ***P < 0.001; ****P < 0.0001.

To elucidate UL55’s mechanism of IFN-β suppression, we first assessed its impact on IFN-β promoter activation using dual-luciferase reporter assays. The results showed that UL55 protein significantly inhibited the activation of IFN-β promoter induced by Poly(I:C) (Fig. 4A), demonstrating targeted suppression of interferon signaling. Given the pivotal role of pattern recognition receptors (PRRs) in initiating IFN-I responses, we systematically evaluated UL55’s effect on key upstream signaling nodes. Co-transfection assays revealed that UL55 broadly suppressed IFN-β promoter activation induced by cGAS, STING, MDA5, RIG-I, MAVS, TBK1, and IRF7 (Fig. 4B through H), indicating that UL55 protein significantly inhibited IFN-β promoter activation mediated by cGAS-STING and RIG-I/MDA5 signaling components under conditions of pathway stimulation.

**Fig 5:**
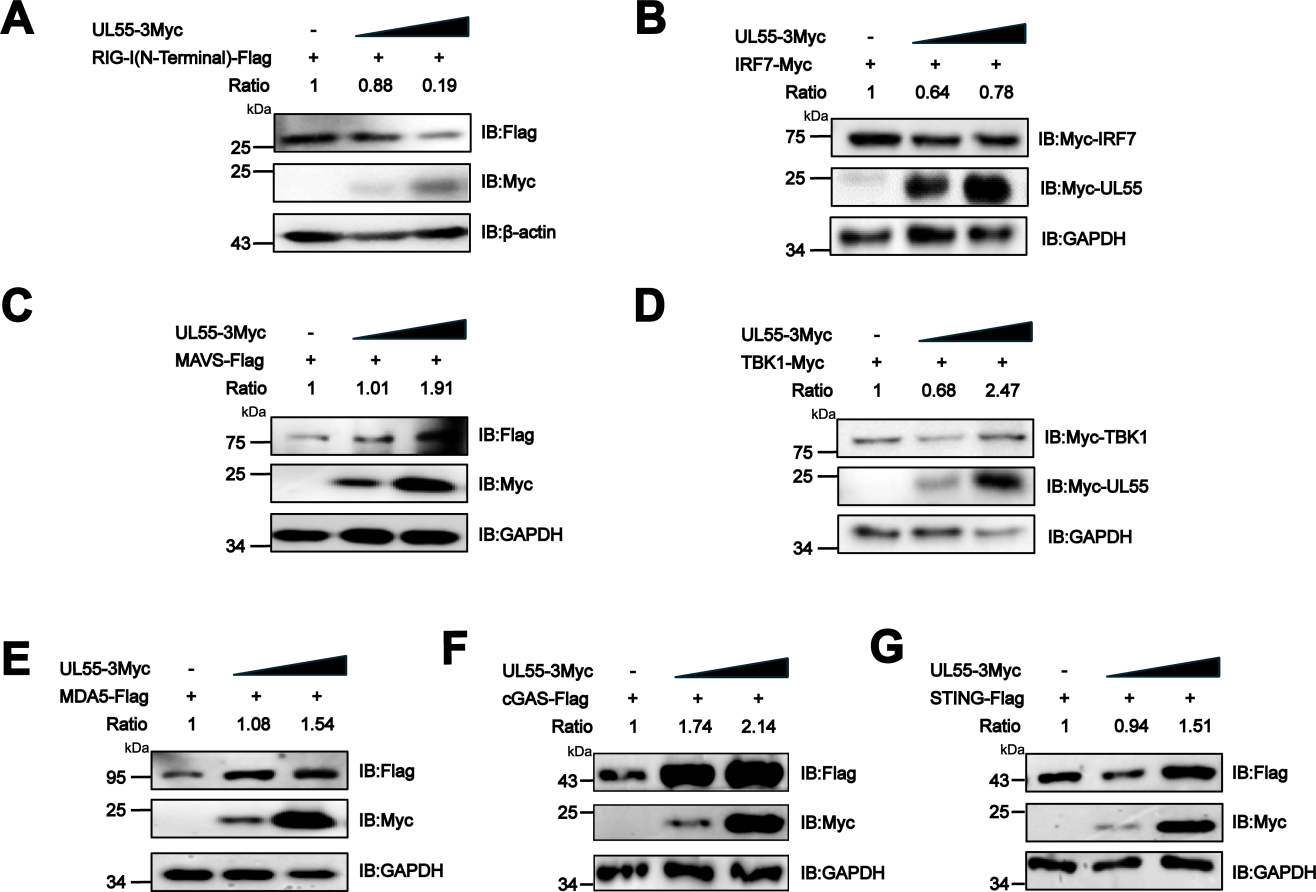
UL55 selectively suppresses RIG-I and IRF7 expression. (**A–G**) Dose-dependent effect of UL55 on innate immune signaling proteins. DEFs were co-transfected with 400 ng of plasmids expressing pCAGGS-RIG-I(N-terminal)-Flag, pCAGGS-MDA5-Flag, pCAGGS-MAVS-Flag, pCAGGS-TBK1-Myc, pCAGGS-STING-FLAG, pCAGGS-cGAS-FLAG, or pCAGGS-IRF7-Myc, alongside increasing concentrations of pCAGGS-UL55-3Myc (0, 300, 600 ng) balanced with empty pCAGGS vector (600, 300, 0 ng) for constant total DNA. Expression levels of pathway proteins were analyzed by Western blotting 36 h post-transfection and quantified by Image J.

This pan-pathway inhibition suggested UL55 targets convergent signaling hubs. To identify specific molecular targets, we performed dose-response assays. Co-transfection of gradient UL55 plasmids (0, 500, 1,000 ng) with pathway effectors demonstrated selective suppression: while RIG-I and IRF7 protein expression decreased dose-dependently (Fig. 5A and B), the cGAS, STING, MDA5, MAVS, and TBK1 remained unaffected (Fig. 5C through G). This pathway specificity suggests that UL55 does not function as a global suppressor of innate immunity but rather selectively interferes with RLR-mediated signaling. Critically, this specificity contrasted with ΔUL55 viral phenotypes, where deletion upregulated all pathway components (Fig. 3A through K). These data suggest that viral infection-induced modulation of non-targeted molecules (e.g., cGAS, MAVS) likely results from indirect effects of UL55 deletion rather than direct regulation. We therefore focused subsequent mechanistic studies on RIG-I and IRF7 as primary targets mediating UL55’s immunosuppressive function.

### UL55 degrades RIG-I and IRF7 via the proteasome pathway

**Fig 6:**
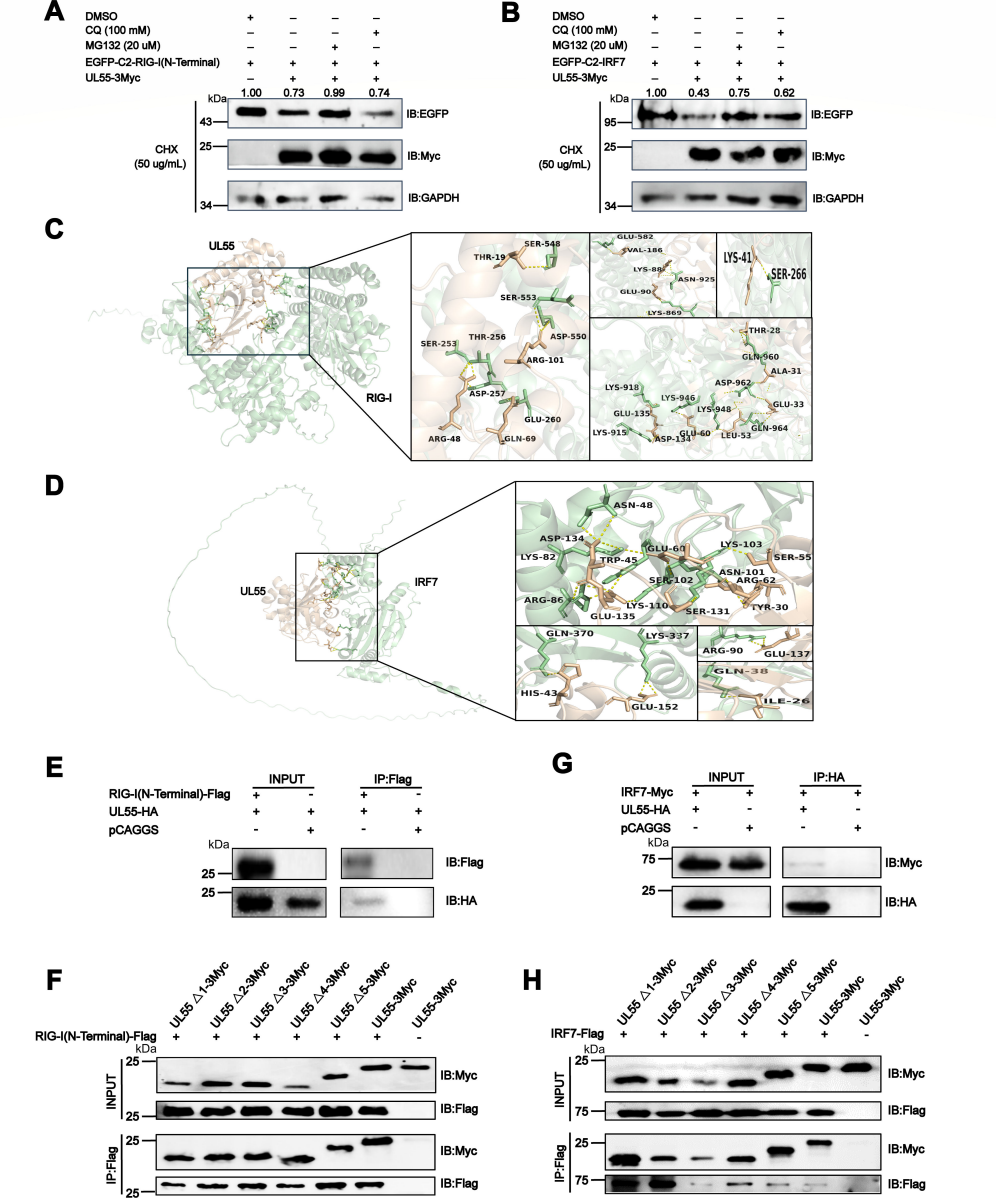
UL55 degrades RIG-I and IRF7 via the proteasome pathway. (**A and B**) Mechanism of UL55-mediated RIG-I/IRF7 degradation. DEFs co-transfected with pCAGGS-UL55-3Myc plus EGFP-C2-RIG-I(N-terminal) (**A**) or EGFP-C2-IRF7 (**B**) were treated at 24 h post-transfection with 50 μg/mL cycloheximide (CHX) combined with either 20 μM MG132 (proteasome inhibitor) or 100 mM chloroquine (CQ, lysosome inhibitor). Cells were harvested after 8 h of treatment. Protein levels were analyzed by Western blotting and normalized to GAPDH. (**C and D**) Structural prediction of UL55 interaction interfaces. Binding interfaces between UL55 and RIG-I(C) or IRF7(D) were computationally modeled using AlphaFold 2. (**E and G**) Confirmation of UL55 binding to RIG-I(NTD) and IRF7. DEFs co-transfected with pCAGGS-UL55-HA or empty vector pCAGGS plus either pCAGGS-RIG-I(N-terminal)-Flag or pCAGGS-IRF7-Myc were harvested at 24 h post-transfection. Anti-Flag (**E**) or anti-HA (**G**) immunoprecipitation followed by Western blotting detected specific interactions. (**F and H**) Mapping of UL55 binding domains. DEFs co-transfected with wild-type/truncated pCAGGS-UL55-HA plasmids plus pCAGGS-RIG-I(N-terminal)-Flag (**F**) or pCAGGS-IRF7-Flag (**H**) were harvested at 24 h post-transfection. Interactions were assessed by anti-Flag immunoprecipitation and Western blotting.

To elucidate UL55’s inhibitory mechanism on RIG-I and IRF7, we first assessed its impact on protein stability. Cycloheximide (CHX, 50 μg/mL) chase assays demonstrated that UL55 co-expression accelerated degradation of RIG-I and IRF7. The quantitative results showing that the degradation of RIG-I by UL55 protein could be significantly rescued by the proteasome inhibitor MG132 (20 μM), indicating that RIG-I is predominantly degraded through the proteasomal pathway (Fig. 6A). For IRF7, however, the analysis revealed that although the lysosome inhibitor chloroquine (CQ, 100 mM) treatment did not restore IRF7 expression to the same extent as MG132 treatment, a partial recovery of IRF7 protein levels was indeed observed upon CQ treatment (Fig. 6B). This suggests that, in addition to the proteasome-dependent pathway, the lysosomal pathway may also contribute to UL55-mediated IRF7 degradation.

This proteasome dependence prompted investigation of molecular interactions. We next performed molecular docking by AlphaFold modeling to predict the potential interaction interface between UL55 and RIG-I/IRF7. The results revealed that UL55 interacts with RIG-I or IRF7 through multiple binding sites (Fig. 6C and D). Co-immunoprecipitation (Co-IP) in DEFs co-expressing full-length UL55 and RIG-I N-terminal domain or IRF7 confirmed the physical binding (Fig. 6E and G). Notably, all UL55 truncation mutants retained binding capacity (Fig. 6F and H), suggesting distributed interaction motifs across UL55’s structure. These findings suggested that UL55 mediated immunosuppression through “molecular interference” of RIG-I or IRF7. Current studies are identifying the E3 ubiquitin ligase mediating this degradation via IP LC-MS/MS analysis.

### UL55 is a conserved IFN-I antagonist with host-adapted mechanisms

**Fig 7:**
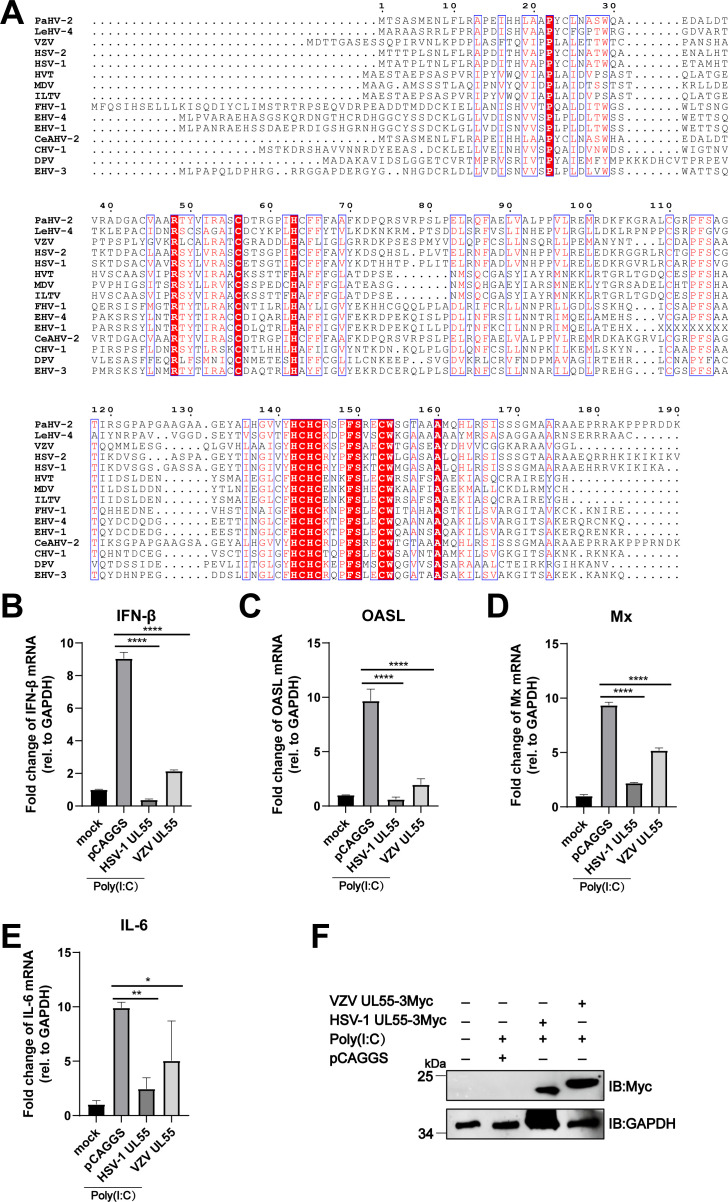
UL55 is a conserved IFN-I antagonist in α-herpesviruses. (**A**) Sequence conservation of UL55 homologs. Representative UL55 sequences from α-herpesviruses (NCBI accession) were aligned using DNAMAN. Red highlights denote identical residues. (**B–F**) HSV-1 and VZV UL55 homologs suppress Poly(I:C)-induced immune responses. DEFs transfected with pCAGGS (vector control), pCAGGS-HSV-1 UL55-3Myc, and pCAGGS-VZV UL55-3Myc were mock-treated or stimulated with Poly(I:C) at 24 h post-transfection for 12 h. mRNA levels of IFN-β (**B**), OASL (**C**), Mx (**D**), and IL-6 (**E**) were quantified by RT-qPCR (18S rRNA-normalized). Expression of HSV-1 and VZV UL55 proteins was confirmed by Western blotting (**F**). Results of the above data were compared using one-way ANOVA. Significance is indicated as follows: ns, not significant; *P < 0.05; **P < 0.01; ***P < 0.001; ****P < 0.0001.

Previously, studies revealed that UL55 homologs are unique to alpha-herpesviruses (28), yet their functional conservation remained unresolved due to low sequence similarity (38.68% amino acid identity; Fig. 7A). To investigate the species-specific mechanisms, we synthesized HSV-1 and VZV UL55 sequences, constructed eukaryotic expression plasmids, and systematically compared the immunomodulatory functions of UL55 derived from different viruses. Functional assays demonstrated that all three homologs could antagonize the production of IFN-β and, therefore, concurrently inhibited IFN-stimulated genes (OASL, Mx) and inflammatory factors (IL-6) induced by Poly(I:C) (Fig. 7B through F). These findings establish UL55 as a conserved IFN-I antagonist across alphaherpesviruses.

**Fig 8:**
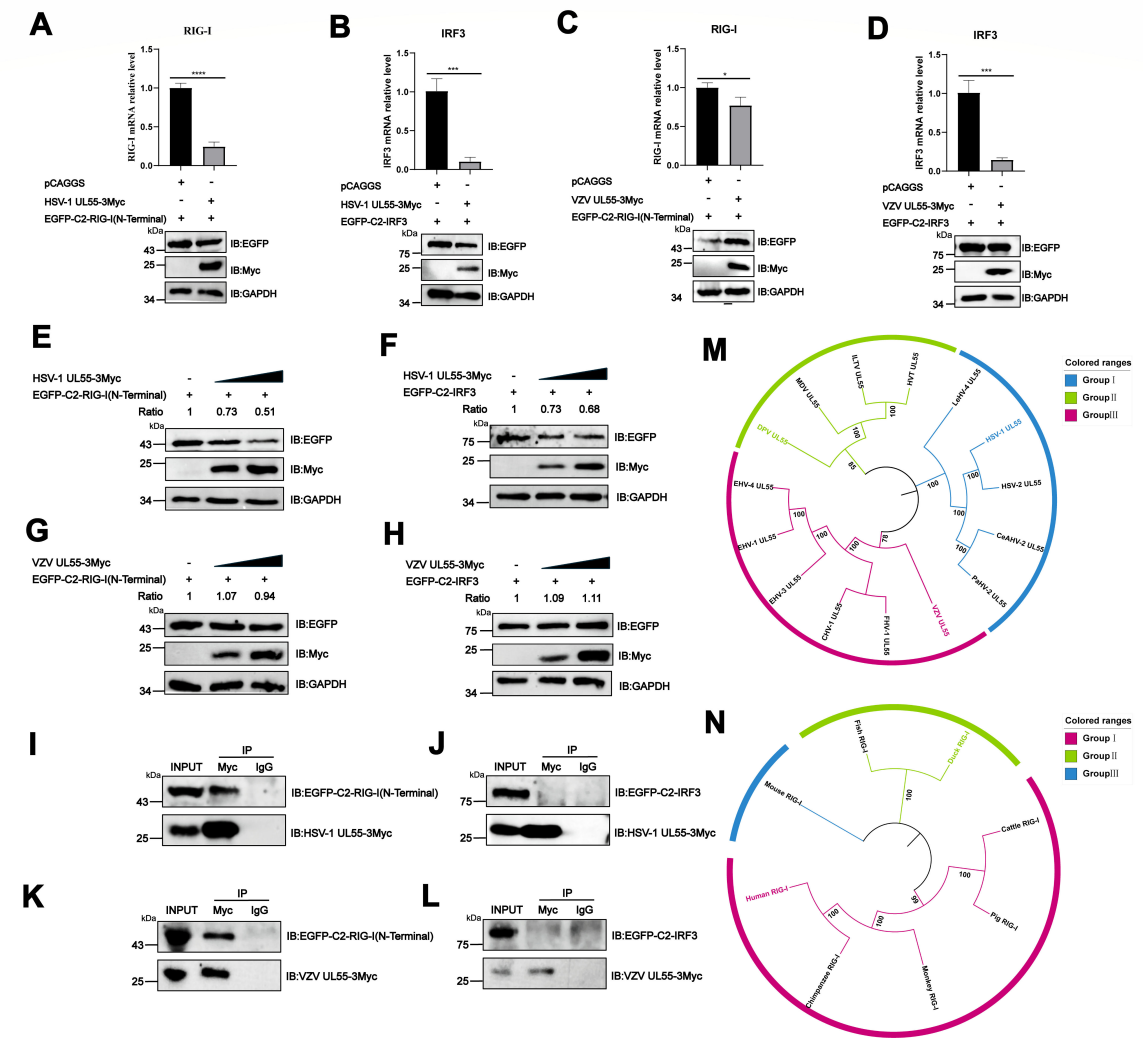
Host-specific mechanism of UL55-mediated immune suppression in alpha herpesviruses. (**A–D**) Transcriptional regulation of RIG-I and IRF3 by HSV-1/VZV UL55. DEFs co-transfected with pCAGGS-HSV-1 UL55-3Myc, pCAGGS-VZV UL55-3Myc, or empty pCAGGS vector alongside EGFP-C2-RIG-I(N-terminal) (**A and C**) or EGFP-C2-IRF3 (**B and D**) were harvested at 36 h post-transfection. mRNA levels of RIG-I (A and C) and IRF3 (B and D) were quantified by RT-qPCR (18S rRNA-normalized). The results were compared using the *t*-test, and the significance analysis is as follows: ns indicates no significant difference; *P* < 0.05; *P* < 0.01; *P* < 0.001; *P* < 0.0001. (**E–H**) Dose-dependent modulation of RIG-I/IRF3 protein expression. DEFs co-transfected with 400 ng EGFP-C2-RIG-I(N-terminal) (**E and G**) or EGFP-C2-IRF3 (**F and H**) plus increasing concentrations of HSV-1/VZV pCAGGS-UL55-3Myc (0/300/600 ng) balanced with pCAGGS vector (600/300/0 ng) were harvested at 36 h. Protein levels were assessed by Western blot. (**I–L**) Interaction analysis of viral UL55 with RIG-I or IRF3. DEFs co-transfected with HSV-1/VZV pCAGGS-UL55-3Myc plus EGFP-C2-RIG-I(N-terminal) (**I and K**) or EGFP-C2-IRF3 (**J and L**) were harvested at 24 h. Anti-Myc immunoprecipitation (vs anti-IgG control) followed by Western blotting detected interactions. (**M and N**) Phylogenetic analysis. Evolutionary trees of alphaherpesvirus (**M**) UL55 and (**N**) RIG-I(N-terminal) amino acid sequences. Branch labels indicate bootstrap values.

Mechanistic analyses revealed a pattern combining both conservation and divergence among UL55 homologs. qPCR results showed that, similar to DPV UL55, UL55 proteins from HSV-1 and VZV significantly reduced the mRNA levels of RIG-I (N-terminal) and IRF3 following transfection (Fig. 8A through D). However, distinct regulatory patterns were observed at the protein level. Consistent with DPV UL55, HSV-1 UL55 induced a dose-dependent reduction in RIG-I and IRF3 protein levels (Fig. 8E and F). In contrast, VZV UL55 did not affect the protein abundance of RIG-I or IRF3 despite reducing their mRNA levels (Fig. 8G and H), suggesting the involvement of post-transcriptional regulatory mechanisms. Co-immunoprecipitation (Co-IP) assays further demonstrated that both HSV-1 and VZV UL55 proteins were able to associate with RIG-I (N-terminal), as RIG-I was detected in UL55-enriched samples (Fig. 8I and K). However, no association with IRF3 was detected (Fig. 8J and L). In comparison, DPV UL55 was able to associate with both RIG-I and IRF7. Together, these results indicate that RIG-I represents a conserved target of UL55 across alpha-herpesviruses, whereas regulation of IRF3/IRF7 exhibits clear divergence among viral species. These findings suggest that UL55 homologs employ both conserved and species-specific strategies to modulate host innate immune signaling.

The subsequent evolutionary analysis revealed the molecular basis of the host-specific immunosuppressive mechanism exerted by the UL55 homolog. Phylogenetic analysis showed UL55 sequences formed distinct clades (bootstrap = 85; Fig. 8M) with DPV clustering closer to HSV-1 than VZV, potentially explaining their functional convergence in IRF3/IRF7 suppression. Host RIG-I exhibited high sequence conservation (78.79%; Fig. S4) but significant phylogenetic divergence between avian and mammalian lineages (Fig. 8N). Critically, host immune architecture differences further shaped this adaptation: waterfowl lack IRF3 and rely solely on IRF7 for IFN-I responses, whereas mammals utilize both IRF3 and IRF7. Therefore, we speculated that alphaherpesviral UL55 achieves immune evasion by targeting the conserved RIG-I molecule while adopting species-specific mechanisms to regulate IRF3/IRF7 pathways. This duality reflects evolutionary optimization at the virus-host interface, where conserved targeting of essential immune sensors coexists with lineage-specific adaptations to host molecular repertoires.

## DISCUSSION

**Fig 9:**
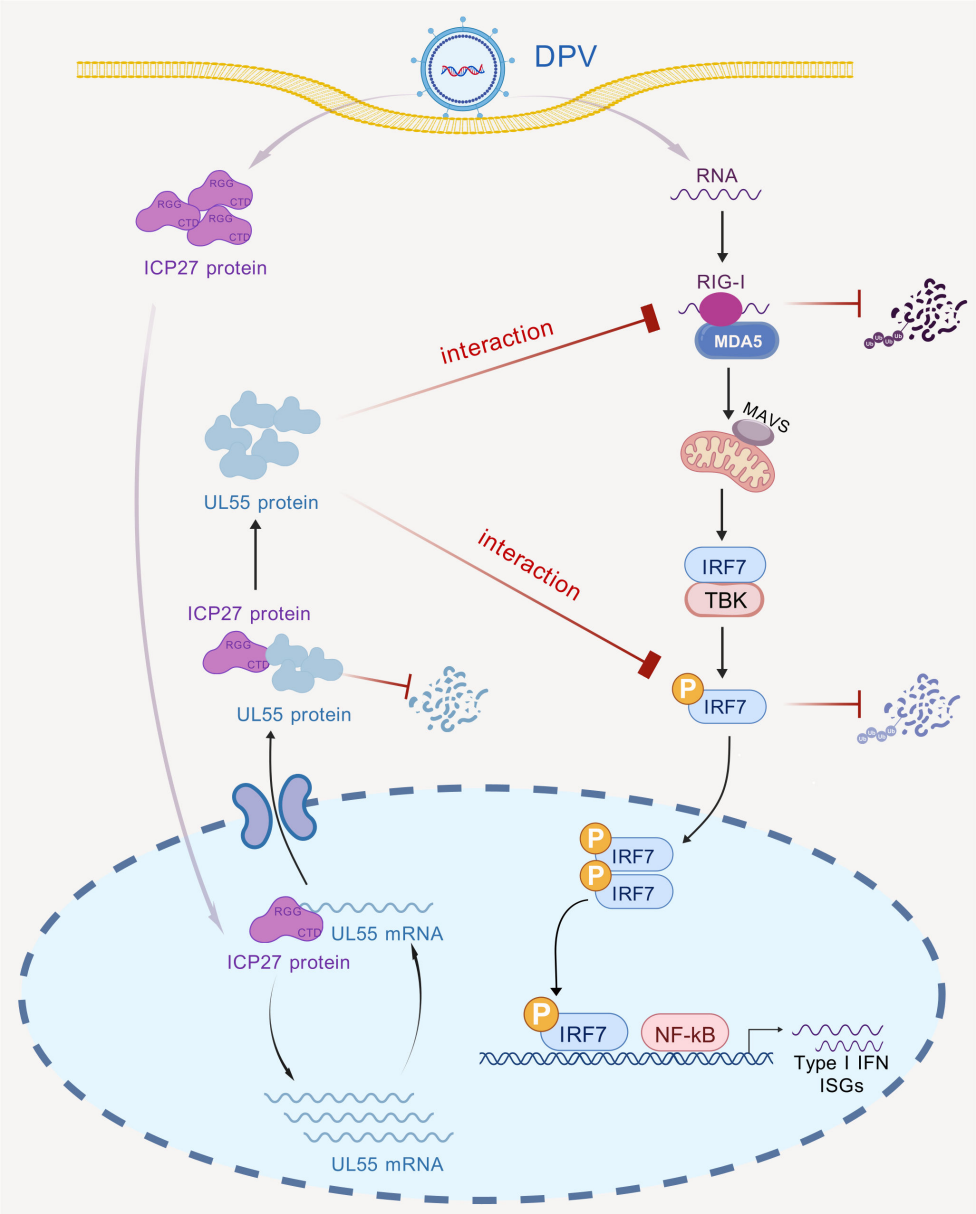
Proposed model of ICP27-UL55 synergistic immune evasion in DPV. ICP27 acts as the master regulator of UL55, orchestrating its functions both transcriptionally and post-translationally. UL55 antagonizes IFN-β production by mediating proteasomal degradation of RIG-I and IRF7. ICP27 amplifies this IFN-I suppression effect through synergistic coordination with UL55. The figure model was created with BioGDP.com (46).

Herpesviruses have evolved sophisticated strategies to counteract host innate immunity for establishing persistent infections (1, 11, 12, 15, 16, 37–39), with duck plague virus (DPV) providing a compelling model for uncovering novel immune evasion paradigms. Our study elucidates a cooperative regulatory axis wherein ICP27 orchestrates UL55-mediated immunosuppression through dual mechanisms (Fig. 9): its RNA-binding domain (RGG) facilitates UL55 mRNA nuclear export, while its C-terminal domain (CTD) stabilizes UL55 protein via physical interaction. This regulatory synergy expands ICP27’s functional repertoire beyond its conserved roles in RNA processing (24–27, 40, 41) and intrinsic IFN-I suppression (unpublished data), positioning it as a master coordinator of immune evasion effectors. Crucially, ICP27’s CTD-mediated stabilization of UL55 represents a distinct functional innovation compared with HSV-1 ICP27, which primarily regulates host gene expression by influencing RNA splicing and polyadenylation (e.g., microtubule-associated protein Tau) (42, 43). The dose-dependent amplification of UL55 by ICP27 reflects evolutionary tuning to balance immunosuppressive potency with host proteostatic constraints—a strategy convergent with HSV-1 ICP34.5’s regulated translation but mechanistically unique (44, 45).

UL55 functions as a precision-targeted IFN-I antagonist, selectively degrading RIG-I and IRF7 mainly through proteasomal pathways while sparing upstream sensors (MDA5, cGAS) and adaptors (MAVS). This targeted degradation strategy minimizes immune activation while crippling key IFN amplification nodes—RIG-I for viral RNA sensing and IRF7 for late-phase IFN production. Mechanistically, UL55’s proteasomal degradation of RIG-I/IRF7 diverges from KSHV vIRF1’s indirect inhibition of IRF3/IRF7 through blocking the signal pathway complex (47, 48), but similarly to PRV US3 directly degrades IRF3 (49), representing a diverse host-specific mechanism in herpesviral immunosuppression.

While cGAS is a central sensor of cytosolic DNA, increasing evidence suggests that RIG-I-like receptors (RLRs) also play important roles in sensing herpesvirus infection. During lytic replication, herpesviruses generate abundant immunostimulatory RNA species, including overlapping transcripts and aberrant RNAs, which can form double-stranded RNA structures and activate RIG-I signalingv (8–10). In this context, RIG-I represents a biologically relevant target for immune evasion, even for DNA viruses. Notably, multiple herpesvirus proteins have been reported to antagonize the RLR pathway. In DPV, we and others have found that viral proteins such as UL41 (12) and UL7 (50) can modulate RIG-I signaling. Similarly, in other herpesviruses, HSV-1 UL37 (51) and KSHV ORF64 (52) have been shown to interfere with RIG-I-mediated antiviral responses, while EBV BPLF1 can simultaneously suppress both RIG-I and cGAS–STING pathways (53). These observations suggest that targeting RNA-sensing pathways is a common and conserved strategy among herpesviruses.

Meanwhile, numerous viral proteins have also been reported to inhibit the cGAS–STING pathway at different levels (19, 54). Therefore, UL55-mediated targeting of RIG-I likely represents a complementary immune evasion strategy, enabling the virus to coordinately suppress parallel sensing pathways and enhance overall inhibition of type I interferon responses. In addition, RIG-I signaling functions as a rapid and amplification-prone component of innate immunity, and its inhibition may efficiently dampen downstream interferon responses. The observation that UL55 homologs from multiple alphaherpesviruses share this activity further supports the idea that targeting RIG-I is an evolutionarily conserved mechanism of immune evasion.

Evolutionarily, UL55 homologs exhibit a conserved divergent functional landscape. All alphaherpesvirus UL55 variants (DPV, HSV-1, VZV) directly bind and suppress RIG-I—a vulnerability conserved across vertebrates (78.79% sequence identity). In contrast, IRF3/IRF7 regulation displays host-adapted divergence: DPV UL55 degrades duck IRF7 (functionally substituting for IRF3 in IRF3-deficient waterfowl); HSV-1 UL55 degrades human IRF3; VZV UL55 spares IRF3 protein but suppresses its transcription. This gradient mirrors fundamental host biology: waterfowl lack IRF3 and rely solely on IRF7 for IFN-I responses, while mammals utilize IRF3/IRF7 redundancy. UL55’s low sequence conservation (38.68% identity) versus RIG-I’s high conservation reflects asymmetric evolutionary pressures—viral effectors rapidly adapt to host-specific defenses while immune sensors maintain cross-species functionality. Phylogenetic clustering of DPV-HSV-1 UL55 (bootstrap = 85) suggests shared evolutionary drivers for IRF3/IRF7 suppression.

The ICP27-UL55 axis exemplifies a coordinated effector-regulation model—a strategy optimizing immune evasion through centralized control. ICP27 fine-tunes UL55 abundance and activity via RGG-mediated mRNA export and CTD-dependent stabilization. This model offers evolutionary advantages: amplification efficiency (one regulator controls effector output), responsive adaptation (rapid tuning to immune pressure), and resilience (conserved RGG domains anchor novel functions). Analogous systems exist—HSV-1 ICP34.5 stabilizes ICP0 (55, 56)—but with distinct mechanisms, indicating convergent evolution across *Herpesviridae*.

Translational opportunities arise from disrupting this axis. Small molecules blocking ICP27 CTD-UL55 80-116AA interaction could attenuate DPV virulence, while UL55-deficient strains (ΔUL55) induce robust IFN responses, serving as vaccine candidates. Future work should identify E3 ligases recruited by UL55, elucidate IRF7 neo-functionalization in IRF3-deficient birds, and explore conservation in related avian herpesviruses (e.g., GaHV-2). These efforts will illuminate fundamental virus-host coevolution principles while advancing strategies against economically devastating avian pathogens.

### Conclusions

This study unveils a layered immune evasion strategy in DPV centered on the ICP27-UL55 partnership. ICP27 acts as a “molecular amplifier,” binding UL55 mRNA via its RGG domain to enhance nuclear export while stabilizing UL55 protein through CTD interaction. UL55 functions as a multi-target IFN-I antagonist, selectively degrading RIG-I and IRF7 via proteasomal pathways. This partnership enables synergistic suppression exceeding individual effects. Evolutionarily, UL55 homologs conserve RIG-I targeting but diverge in IRF3/IRF7 regulation, reflecting host-specific adaptations shaped by UL55 sequence variation and host transcription factor repertoires. This work redefines herpesviral immune evasion through an “effector-coordination model,” wherein ICP27 optimizes UL55 immunosuppression while maintaining intrinsic IFN-I suppression. Disrupting this axis offers new antiviral strategies, with UL55-deficient strains serving as vaccine candidates for poultry disease control.

## MATERIALS AND METHODS

### Cells and viruses

Duck embryo fibroblasts (DEFs), BHK-21, and HEK-293T cells were cultured and maintained in Dulbecco’s modified Eagle’s medium (DMEM) (Servicebio, Wuhan, China, G4524-500ML) supplemented with 10% fetal bovine serum (FBS) (Gibco Life Technologies, Shanghai, China, A5256701). For viral infections or plasmid transfections, the medium was supplemented with 2% FBS. All cells were cultured in 6-, 12-, or 24-well plates (LABSELECT, Beijing, China) at 37°C in a 5% CO₂ atmosphere.

The wild-type duck plague virus (DPV) CHv (GenBank Accession No. JQ647509.1), the parental virus DPV CHv-BAC, and recombinant viruses DPV CHv-BAC-ΔICP27 (57) and DPV CHv-BAC-ΔUL55 (28) were generated and maintained in our laboratory. Virus stocks were prepared and titrated using the Reed-Muench assay on DEF cells.

### Antibodies and key reagents

The following primary antibodies were used: anti-FLAG antibody (ABmart, Beijing, China, M20008M; Proteintech, Wuhan, China, 66008-4-Ig; Yeasen, Shanghai, China, 30503ES60), anti-HA antibody (Proteintech, 51064-2-AP; ABmart, M20003M), anti-GFP antibody Mouse mAb (ABmart, M20004M), anti-Myc antibody (Abclonal, Wuhan, China, AE070; Proteintech, 60003-2-Ig), anti-GAPDH (Proteintech, 60004-1-Ig), anti-β-actin Rabbit antibody mAb (Abclonal, AC038), anti-Lamin B1 antibody (ABmart, P20700), rabbit control IgG (Abclonal, AC005), mouse control IgG (Abclonal, AC011), anti-ICP27, UL55, and ICP8 polyclonal antibody were prepared in our laboratory. The secondary antibodies were as follows: HRP-conjugated goat anti-mouse IgG (H + L) (SA00001-1) and HRP-conjugated goat anti-rabbit IgG (H + L) (SA00001-2) were purchased from Proteintech. The other reagents used and their sources were as follows: Transfection Reagent Lipo2000 (Lab, Beijing, China, TR001), TransDetect Double-Luciferase Reporter Assay Kit (TransGen Biotech, Beijing, China, FR201-02), RNA-easyTM Isolation Reagent (Vazyme, Nanjing, China, R711), First-strand cDNA Synthesis Mix (Lab, F0202), 2× Realab Green PCR Fast mixture (Lab, R0202), protein A/G Magnetic Beads (MedChemExpress, USA, HY-K0202), Cycloheximide (CHEJETER, Beijing, China, 66-81-9), Actinomycin D (MedChemExpress, HY-17559), Poly(I:C) (InvivoGen, France, tlrl-pic), 2× In-Fusion Cloning Mix (Servicebio, G3350), Clarity Western ECL substrate (Bio-Rad, 1705061), NucleoBond Xtra Midi kit for transfection-grade plasmid DNA (MACHEREY-NAGEL, Germany, 740410.5), RIPA (Lab, R1091), IP (Lab, W0013), and nucleocytoplasmic separation kit (Beyotime, Shanghai, China, P0027).

### Plasmid construction

The coding sequences for DPV ICP27 and UL55 were amplified from viral genomic DNA and cloned into the pCAGGS vector with corresponding tags using the 2× In-Fusion Cloning Mix. Site-directed mutagenesis was employed to introduce specific mutations into the targeted regions of ICP27 and UL55 following the same protocol. HSV-1 UL55 (accession no. 2703427) and VZV UL55 (accession no. 1487681) sequences were synthesized and cloned into the pCAGGS vector by Sangon Biotech (Shanghai, China). The human RIG-I (accession no. 23586) and IRF3 (accession no. 3661) sequences were amplified from 293T cells and cloned into EGFP-C2. All plasmids were verified by DNA sequencing. The plasmids related to duck immune responses, such as pCAGGS-cGAS-Flag, pCAGGS-cGAS-HA, pCAGGS-STING-Flag, pCAGGS-RIG-I(N-Terminal)-Flag, EGFP-C2-RIG-I(N-Terminal), pCAGGS-MDA5-Flag, pCAGGS-MAVS-Flag, pCAGGS-TBK1-Flag, pCAGGS-TBK1-Myc, pCAGGS-IRF7-Flag, pCAGGS-IRF7-Myc, EGFP-C2-IRF7, and pGL-Du IFN-β PRO, were generated by amplifying the CDS sequences of the corresponding genes from the duck genome, and then ligating them to the appropriate vectors (pCAGGS, EGFP, or pGL, etc.) according to the instructions of the ClonExpress II One Step Cloning Kit (Vazyme, Cat. No: C112-01). The corresponding tags (FLAG, HA, MYC, etc.) were fused at the C-terminal during plasmid construction. All plasmids were verified to be correct by PCR and sequencing, and were then stored in our laboratory. The pRL-TK internal reference vector and pGL4.10 vector were purchased from Promega.

### Transfection and overexpression

DEF, BHK-21, and HEK-293T cells were transfected with the relevant plasmids using Transfection Reagent Lipo2000 according to the manufacturer’s instructions. In dose-dependent experiments, increasing amounts of the ICP27 plasmid were co-transfected with a constant amount of the UL55 plasmid. Similarly, increasing amounts of the UL55 plasmid were co-transfected with constant amounts of the cGAS, STING, RIG-I, MDA5, MAVS, TBK1, or IRF7 plasmids.

### Western blotting analysis and co-immunoprecipitation (Co-IP)

DEF, BHK-21, or HEK-293T cells in six-well plates were infected with viruses or transfected with plasmids and harvested at the indicated time points. Mock-infected or empty vector-transfected cells were used as controls. Samples were lysed in RIPA buffer with protease inhibitors, proteins separated on 12% SDS-PAGE, and transferred to PVDF membranes. Membranes were blocked with 5% skim milk, then incubated overnight with corresponding primary antibodies. Secondary antibodies were applied for 1 hour at 37°C. Proteins were detected using ECL substrate, and band intensities were analyzed with ImageJ. For protein–protein interaction studies, DEF, BHK-21, or HEK-293T cells co-expressing tagged versions of proteins were lysed in IP buffer. Lysates were incubated with tagged antibodies against ICP27, UL55, RIG-I, or IRF7, and protein complexes were captured with protein A/G beads. After washing, the immunoprecipitates were subjected to 12% SDS-PAGE and Western blot analysis. Domain mapping was performed using deletion mutants of ICP27 and UL55 to identify regions critical for interaction.

### Immunoprecipitation and LC–MS/MS analysis

Cells were infected with 1 MOI ICP27-Flag–tagged DPV or control DPV virus and harvested at 24 h post-infection. Cell lysates were prepared using IP lysis buffer. After clarification by centrifugation, supernatants were incubated with an Anti-FLAG antibody at 4°C overnight, followed by immunoprecipitation using Protein A/G beads. The beads were stringently washed with ice-cold TBS buffer. The resulting protein complexes were sent to a commercial proteomics service provider (Shanghai OE Biotech Co., Ltd.) for the subsequent liquid chromatography–tandem mass spectrometry (LC–MS/MS) analysis. Peptide separation and MS/MS data acquisition were carried out using the technical platform based on the Easy-nLC 1200 ultra-high performance liquid chromatography and the Q Exactive high-resolution mass spectrometer. Combined with the search library software (ProteomeDiscover 2.5 software), it conducted protein qualitative (or relative quantitative) analysis on samples from different experimental groups. Protein identification was filtered at a false discovery rate (FDR) of <1% at both peptide and protein levels. Candidate interacting proteins were selected based on their enrichment in ICP27-Flag immunoprecipitates relative to control samples, rather than peptide abundance alone. The list of proteins obtained in the experiment can reflect the types and relative contents of proteins detected by the mass spectrometry.

### Quantitative real-time PCR (RT-qPCR)

Total RNA was extracted from cells infected with viruses or transfected with plasmids using RNA-easy Isolation Reagent. cDNA was synthesized using First-strand cDNA Synthesis Mix. Target genes were detected using specific primers (Table S2), and 18S rRNA was used as a reference gene for normalization. All reactions were performed in triplicate with at least three independent experiments. The relative gene expression levels were determined using the 2**^−^**^ΔΔCt^ method.

### Nuclear-cytoplasmic fractionation and mRNA stability assays

For nuclear export analysis, the DEF cells inoculated with 1 MOI DPV-△ICP27 or DPV-CHv for 12 h were harvested and fractionated into nuclear and cytoplasmic compartments using a commercial kit. UL55 mRNA levels in each fraction were quantified by RT-qPCR. Lamin B1 and GAPDH were used for nuclear and cytoplasmic markers. To assess mRNA stability, cells infected with 1 MOI wild-type DPV (DPV-WT) or ICP27 deletion mutant (DPV-△ICP27) were treated with Actinomycin D (2 µg/mL) at 24 hpi, and total RNA was harvested at the indicated time points for RT-qPCR analysis. Each of these experiments was performed with three independent biological replicates.

### Protein stability assay

Protein stability was assessed using cycloheximide (CHX) chase assays. DEF cells transfected with UL55, with or without ICP27, were treated with CHX (50 µg/mL) at 24 h post-transfection to inhibit protein synthesis. Cells were harvested at various time points post-treatment, and UL55 protein levels were determined by Western blotting.

### RNA immunoprecipitation (RIP) assay

RNA immunoprecipitation (RIP) was performed using an anti-ICP27 tagged antibody to investigate its interaction with UL55 mRNA, followed by agarose gel electrophoresis analysis of the precipitated RNA. Specifically, DEF cells were transfected with wild-type or mutant ICP27 plasmids 12 h before infection with 1 MOI of DPV-ΔICP27. At 24 hpi, cells were lysed in IP buffer and incubated on ice for 30 min. Lysates were centrifuged at 10,000 rpm for 10 min at 4°C, and 50 µL of the supernatant was saved as the input sample and stored at −20°C. The remaining lysate was incubated with an anti-HA antibody or control IgG for 8h at 4°C, and the immunocomplexes were captured using protein A/G magnetic beads. After a 2-h incubation at 4℃, the bead-antibody-protein/RNA complexes were washed three times with PBST. Finally, the bead-antibody-protein/RNA complexes were suspended in 150 µL of DEPC-treated water, and 50 µL was used for IP histone analysis by Western blot, using the input group as a control. RNA from the remaining samples was extracted using First-strand cDNA Synthesis Mix and analyzed by agarose gel electrophoresis to quantify UL55 mRNA levels.

### Dual-luciferase reporter assay

Promoter activation assays were conducted by co-transfecting DEF cells with luciferase reporter constructs driven by IFN-β upstream promoters and specific expression vectors. Cells were harvested 36 h post-transfection (hpt.), and luciferase activity was measured using the dual-luciferase reporter assay system, with Firefly luciferase activity normalized to Renilla luciferase activity. Each of these experiments was performed with three independent biological replicates.

### Protein degradation assays

This assay was conducted in duck embryo fibroblasts (DEFs) co-transfected with pCAGGS-UL55-3Myc and either EGFP-C2-RIG-I (N-terminal) or EGFP-C2-IRF7 plasmids using transfection reagent Lipo2000. At 24 h post-transfection, cells were treated with 50 μg/mL cycloheximide (CHX) combined with either 20 μM MG132 (proteasome inhibitor) or 100 mM chloroquine (CQ, lysosome inhibitor). Cells were harvested at 8 h post-treatment for Western blot analysis using anti-GFP (detecting RIG-I/IRF7 fusion proteins), anti-Myc (detecting UL55), and anti-GAPDH antibodies. Protein levels were quantified through grayscale analysis of band intensities normalized to GAPDH using ImageJ software.

### Homology and evolutionary analysis

To investigate the homology and evolutionary relationships of UL55 and RIG-I proteins across different species, we conducted sequence alignment and phylogenetic analysis using MEGA 7.0. The evolutionary history was inferred using the neighbor-joining method, and sequence alignments were visualized with ESPript 3.0.

For UL55, all amino acid sequences from α-herpesviruses were retrieved from NCBI: DPV UL55 (YP_003084363.1), PaHV-2 UL55 (YP_443903.1), LeHV-4 UL55 (YP_009230190.1), VZV UL55 (XPC70989.1), HSV-2 UL55 (YP_009137208.1), HSV-1 UL55 (WWU03448.1), HVT UL55 (NP_066889.1), MDV UL55 (YP_001033986.1), ILTV UL55 (BAA33006.1), FHV-1 UL55 (YP_003331523.1), EHV-4 UL55 (NP_045221.1), EHV-1 UL55 (QXN54612.1), CeAHV-2 UL55 (YP_164499.1), CHV-1 UL55 (YP_009252230.1), and EHV-3 UL55 (YP_009054907.1).

For RIG-I, amino acid sequences from eight species were obtained: Duck RIG-I (NP_001297309.1), Human RIG-I (NP_055129.2), Mouse RIG-I (NP_766277.3), Pig RIG-I (NP_998969.2), Chimpanzee RIG-I (XP_001156662.1), Cattle RIG-I (XP_002689526.3), Monkey RIG-I (NP_001036133.1), and Bird RIG-I (XP_064902334.1). This approach allowed for a detailed comparison of the evolutionary conservation and divergence of these key viral and host proteins.

### Immune response analysis

The impact of UL55 on immune signaling pathways (cGAS-STING, RIG-I/MDA5) was analyzed by qPCR (performed in three independent biological replicates) and Western blot. DEF cells were infected with wild-type or UL55-mutant DPV, and changes in the expression of immune-related genes and proteins were assessed.

### Statistical analysis

All experiments were repeated at least three times independently and analyzed using GraphPad Prism 7.0 software (La Jolla, CA, USA). Results are presented as mean ± SD. Statistical significance was determined using one-way ANOVA, and the statistically significant differences are shown below: *P < 0.05; **P < 0.01; ***P < 0.001; ****P < 0.0001; and ns, not significant.

## Data Availability

This study does not involve any data that has been uploaded to public databases. All original data are available from the authors upon reasonable request.

## SUPPLEMENTAL MATERIAL

Fig. S1 jvi.00653-26-s0001.tif Characterization of DPV-ICP27-Flag recombinant virus and mass spectrometry screening.

Fig. S2 jvi.00653-26-s0002.tif Co-IP validation of host and viral proteins enriched by IP-LC MS.

Fig. S3 jvi.00653-26-s0003.tif ICP22 does not significantly alter UL55 expression.

Fig. S4 jvi.00653-26-s0004.tif Cross-species conservation analysis of RIG-I.

Supplemental Material jvi.00653-26-s0005.docx Legends for Fig. S1 to S3; Table S2.

Table S1 jvi.00653-26-s0006.xlsx Viral and host proteins identified by IP-MS/MS.

ASM does not own the copyrights to Supplemental Material that may be linked to, or accessed through, an article. The authors have granted ASM a non-exclusive, world-wide license to publish the Supplemental Material files. Please contact the corresponding author directly for reuse.
