# Supplementary figures and images for: Alpha-herpesvirus UL55 synergizes with ICP27 to suppress type I interferon production through conserved and host-adapted mechanisms
Source: J Virol. 2026 Jun 30;100(7):e00653-26. doi: 10.1128/jvi.00653-26 (PMC13386910; doi:10.1128/jvi.00653-26)

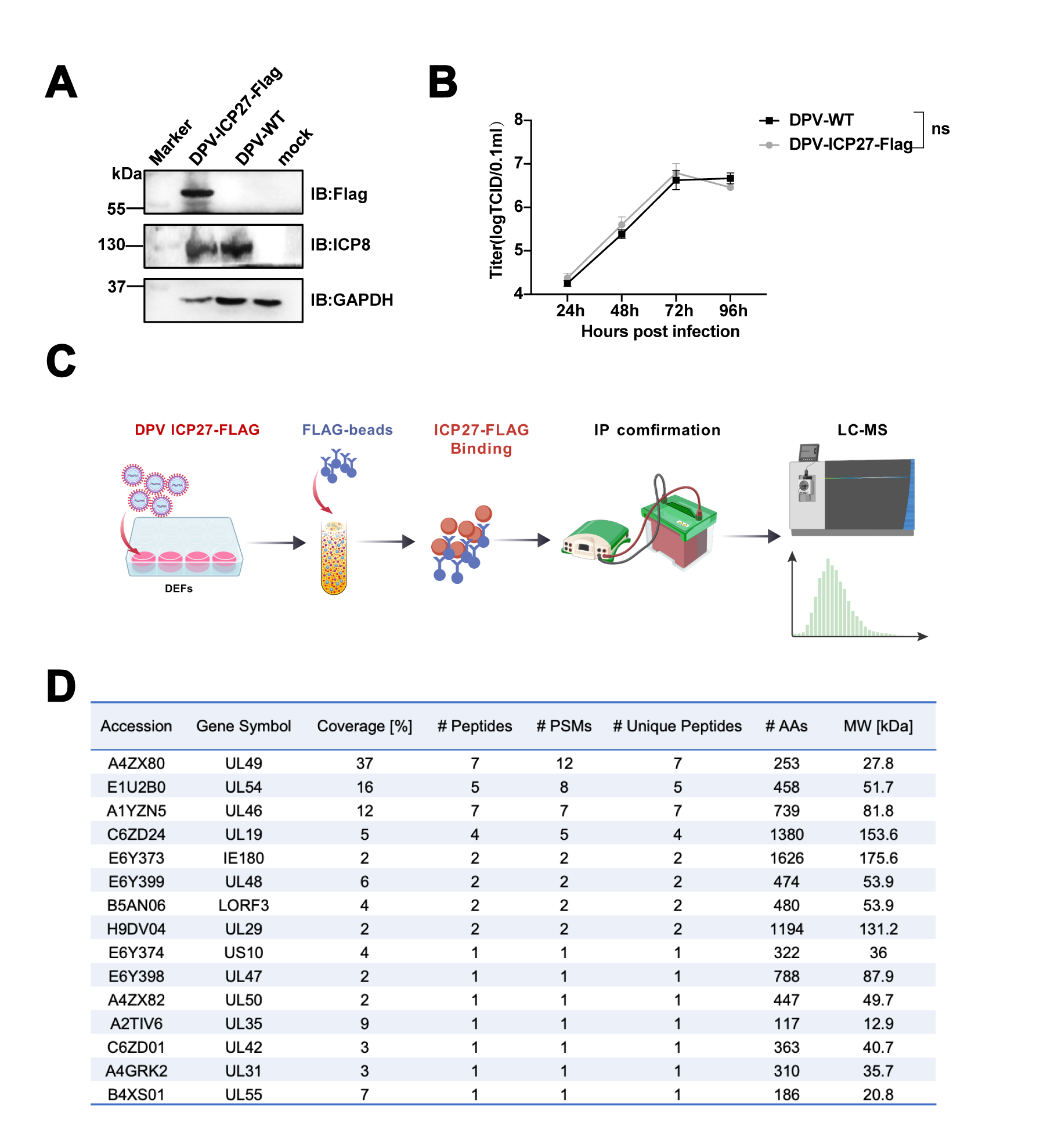

Supplement: Fig. S1 — Characterization of DPV-ICP27-Flag recombinant virus and mass spectrometry screening. [file jvi.00653-26-s0001.tif]

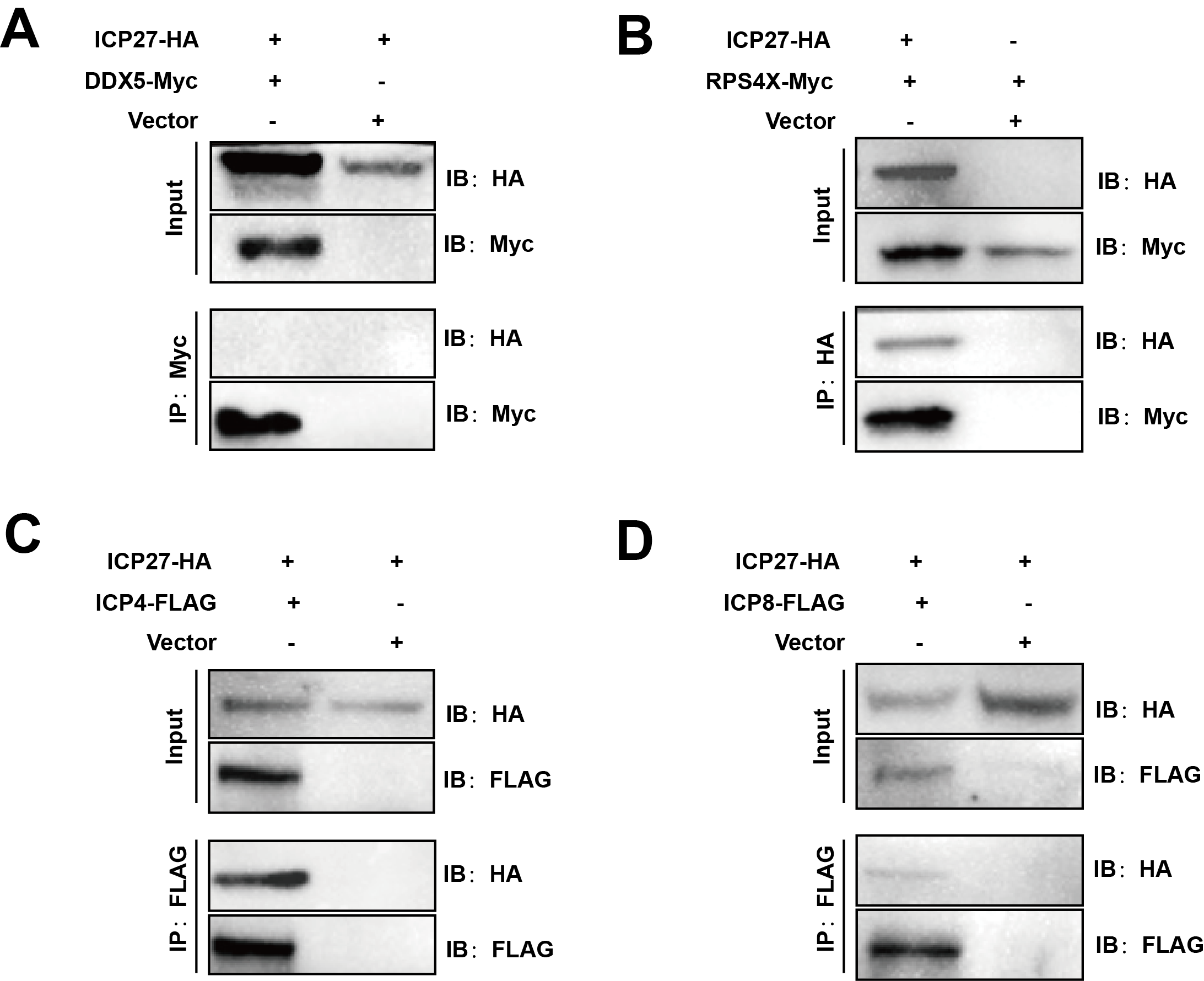

Supplement: Fig. S2 — Co-IP validation of host and viral proteins enriched by IP-LC MS. [file jvi.00653-26-s0002.tif]

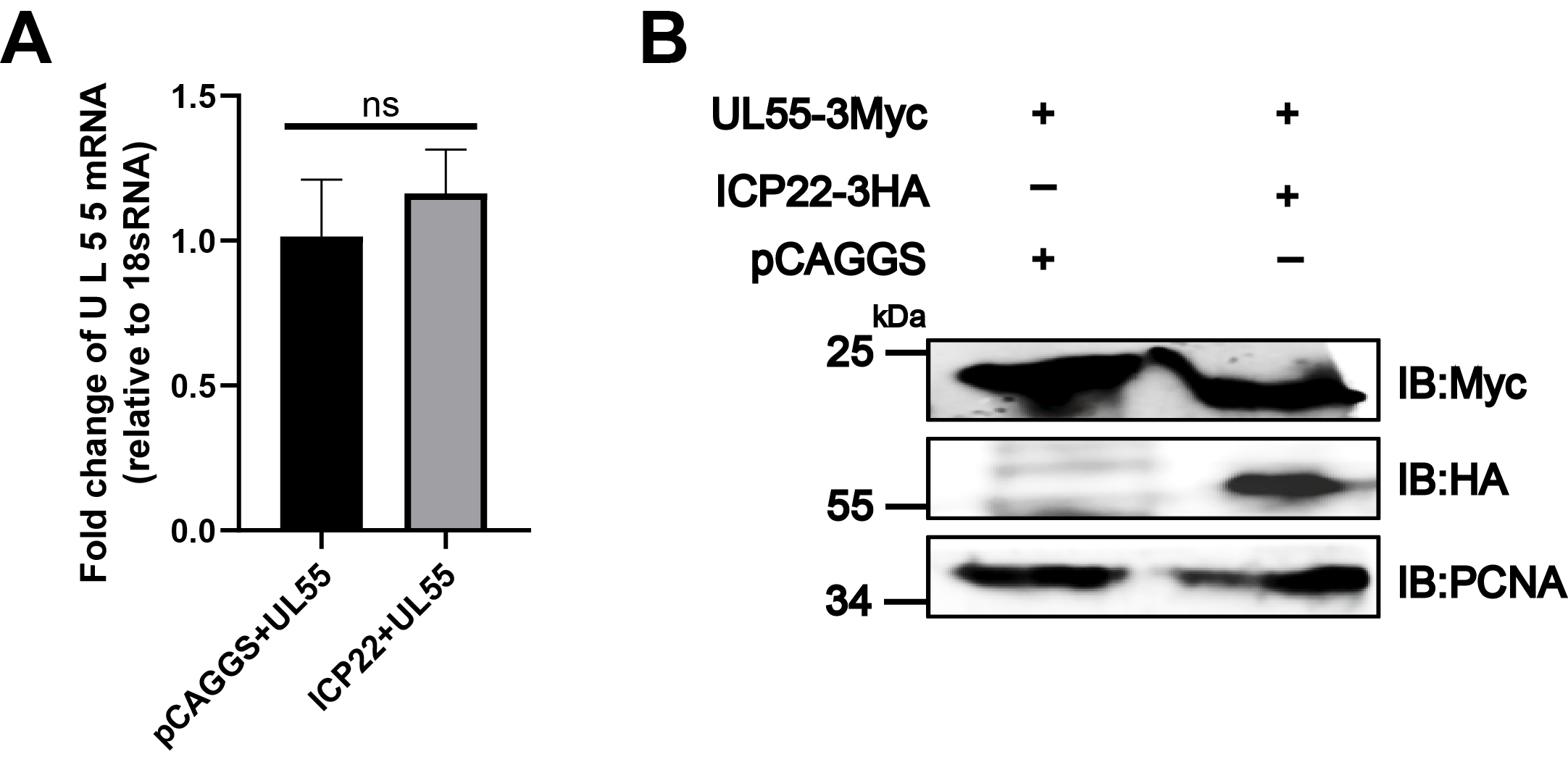

Supplement: Fig. S3 — ICP22 does not significantly alter UL55 expression. [file jvi.00653-26-s0003.tif]

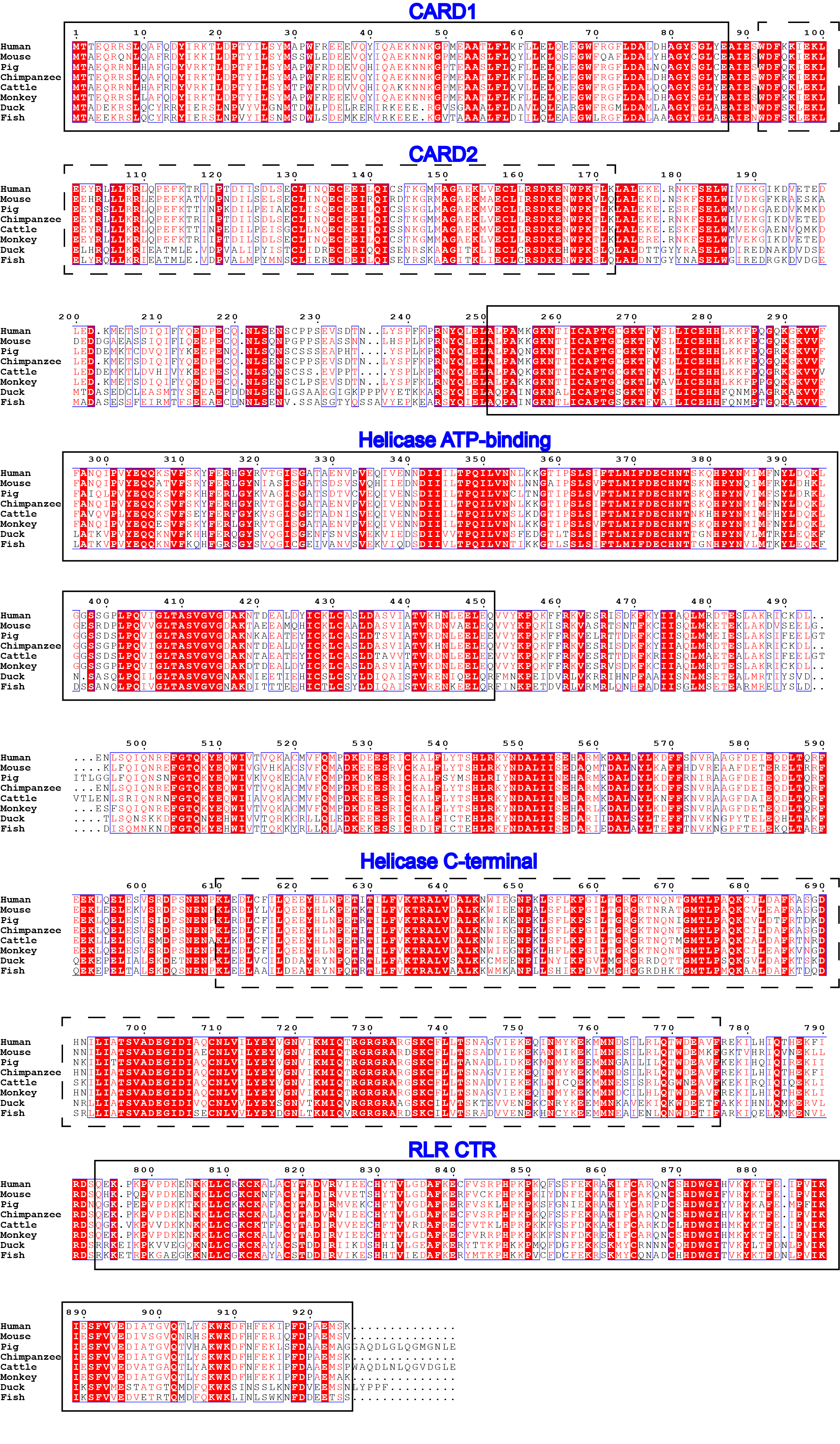

Supplement: Fig. S4 — Cross-species conservation analysis of RIG-I. [file jvi.00653-26-s0004.tif]
