# Supplementary material for: Alpha-herpesvirus UL55 synergizes with ICP27 to suppress type I interferon production through conserved and host-adapted mechanisms
Source: J Virol. 2026 Jun 30;100(7):e00653-26. doi: 10.1128/jvi.00653-26 (PMC13386910; doi:10.1128/jvi.00653-26)
Supplement: Supplemental Material — Legends for Fig. S1 to S3; Table S2. [file jvi.00653-26-s0005.docx]

**SUPPORTING INFORMATION**

**Supplementary Figures**

**Figure S1. Characterization of DPV-ICP27-Flag recombinant virus and mass spectrometry screening.​​**

**(A) Validation of Flag-tagged ICP27 expression.** DEFs infected with DPV-ICP27-Flag or DPV-WT (MOI=1) were harvested at 24 hpi. C-terminal Flag fusion was confirmed by Western blot using anti-Flag antibody. **(B) Replication kinetics.** DEFs infected with DPV-ICP27-Flag or DPV-WT (MOI=0.01). Viral titers at 24, 48, 72, and 96 hpi were determined by TCID_50_ assay(ns: P>0.05 by one-way ANOVA). **(C) IP-LC MS workflow.** DEFs infected with DPV-ICP27-Flag (MOI=1) was harvested at 24 hpi. Anti-Flag immunoprecipitates were analyzed by LC-MS/MS to identify ICP27-interacting proteins. **(D) Candidate ICP27-interacting proteins.** Proteins specifically enriched in DPV-ICP27-Flag immunoprecipitates.

**Figure S2. Co-IP validation of host and viral proteins enriched by IP-LC MS.**

**(A-B)** In DEF cells, the ICP27-HA plasmid and two candidate host protein plasmids (DDX5-myc, RPS4X-myc) screened by IP-LC MS were co-transfected, an empty pCAGGS plasmid were transfected as a vector control for CO-IP verification of the interaction between these two proteins. **(C-D)** In DEF cells, the ICP27-HA plasmid and two candidate viral proteins (ICP4-FLAG, ICP8-FLAG) screened by IP-LC MS were co-transfected, an empty pCAGGS plasmid were transfected as a vector control for CO-IP verification of the interaction between these two proteins.

**Figure S3. ICP22 does not significantly alter UL55 expression.​​**

**(A-B)** DEFs co-transfected with pCAGGS-UL55-3Myc plus pCAGGS-ICP22-3HA or empty pCAGGS vector. Cells were harvested at 24 h post-transfection. UL55 mRNA (A) and protein (B) levels were measured by RT-qPCR and Western blot. **Figure S4. Cross-species conservation analysis of RIG-I.​​**

Representative RIG-I sequences from vertebrates (NCBI accessions) were aligned using DNAMAN. Red shading indicates identical residues across species.

**TABLES**

**Table S2 The sequences of all primers used in this study.**

| Primers | Sequence （5’–3’） |
| --- | --- |
| DPV UL55-3Myc-F | CATCATTTTGGCAAAGAATTCGCCACCATGGCCGACGCGAAGGCG |
| DPV UL55-3Myc-R | AATGAGCTTTTGCTCCTCGAGTACATTAGCTTTGTGAATTCC |
| UL55 Δ1-3Myc-F | CATCATTTTGGCAAAGAATTCGCCACCATGAAGGACCACTGTGTT |
| ∆UL55Δ1-3Myc-R | AATGAGCTTTTGCTCCTCGAGTACATTAGCTTTGTGAATTCC |
| UL55 Δ2-3Myc-F1 | CATCATTTTGGCAAAGAATTCGCCACCATGGCCGACGCGAAGGCG |
| UL55 Δ2-3Myc-R1 | GCATAAAATGAGCCCGCAGAACTTCTTGGGCATGTAAAACAT |
| UL55 Δ2-3Myc-F2 | ATGTTTTACATGCCCAAGAAGTTCTGCGGGCTCATTTTATGC |
| UL55 Δ2-3Myc-R2 | AATGAGCTTTTGCTCCTCGAGTACATTAGCTTTGTGAATTCC |
| UL55 Δ3-3Myc-F1 | CATCATTTTGGCAAAGAATTCGCCACCATGGCCGACGCGAAGGCG |
| UL55 Δ3-3Myc-R1 | ATATGGCGCGTTGCATAGACGGAATATATGTGATTCTGTTTC |
| UL55 Δ3-3Myc-F2 | GAAACAGAATCACATATATTCCGTCTATGCAACGCGCCATAT |
| UL55 Δ3-3Myc-R2 | AATGAGCTTTTGCTCCTCGAGTACATTAGCTTTGTGAATTCC |
| UL55 Δ4-3Myc-F1 | CATCATTTTGGCAAAGAATTCGCCACCATGGCCGACGCGAAGGCG |
| UL55 Δ4-3Myc-R1 | AGCCGATGCCGATACGACCCCATGCTCCGTACGTATTCCGGC |
| UL55 Δ4-3Myc-F2 | GCCGGAATACGTACGGAGCATGGGGTCGTATCGGCATCGGCT |
| UL55 Δ4-3Myc-R2 | AATGAGCTTTTGCTCCTCGAGTACATTAGCTTTGTGAATTCC |
| UL55 Δ5-3Myc-F | CATCATTTTGGCAAAGAATTCGCCACCATGGCCGACGCGAAGGCG |
| UL55 Δ5-3Myc-R | AATGAGCTTTTGCTCCTCGAGCTGCCAACAACTCATTGA |
| HSV-1 UL55-3Myc-F | CATCATTTTGGCAAAGAATTCGCCACCATGACAGCGACCCCCCTC |
| HSV-1 UL55-3Myc-R | AATGAGCTTTTGCTCCTCGAGCGCCTTAATTTTAATCTT |
| ICP27-3HA-F | CATCATTTTGGCAAAGAATTCGCCACCATGGCCTGCAGTGCTAAACCCG |
| ICP27-3HA-R | CACATCATAAGGATAGGTACCAAACATTTCATTACAATAAAAGTACTTCCCGTGC |
| ICP27 ΔN-3HA-F | CATCATTTTGGCAAAGAATTCGCCACCATGAAGAAAAAACCCTCCGATCAT |
| ICP27 ΔN-3HA-R | CACATCATAAGGATAGGTACCAAACATTTCATTACAATAAAAGTACTTCCCGTGC |
| ICP27 ΔNLS1-3HA-F1 | CATCATTTTGGCAAAGAATTCGCCACCATGGCCTGCAGTGCTAAACCCG |
| ICP27 ΔNLS1-3HA-R1 | CCTCGCTCGTTTAACATATTTTTCATTAGCAGTAGTTCCGCG |
| ICP27 ΔNLS1-3HA-F2 | CGCGGAACTACTGCTAATGAAAAATATGTTAAACGAGCGAGG |
| ICP27 ΔNLS1-3HA-R2 | CACATCATAAGGATAGGTACCAAACATTTCATTACAATAAAAGTACTTCCCGTGC |
| ICP27 ΔRGG-3HA-F1 | CATCATTTTGGCAAAGAATTCGCCACCATGGCCTGCAGTGCTAAACCC |
| ICP27 ΔRGG-3HA-R1 | ATTATCAGATTGCATTTTAGACCCAGTATCATGATCGGAGGG |
| ICP27 ΔRGG-3HA-F2 | CCCTCCGATCATGATACTGGGTCTAAAATGCAATCTGATAAT |
| ICP27 ΔRGG-3HA-R2 | CACATCATAAGGATAGGTACCAAACATTTCATTACAATAAAAGTACTTCCCGTGC |
| ICP27 ΔNLS2-3HA-F1 | CATCATTTTGGCAAAGAATTCGCCACCATGGCCTGCAGTGCTAAACCC |
| ICP27 ΔNLS2-3HA-R1 | AGATGCCCATGGGTGTTCGGCTTGATGGCGCTGGTCGTGATC |
| ICP27 ΔNLS2-3HA-F2 | GATCACGACCAGCGCCATCAAGCCGAACACCCATGGGCATCT |
| ICP27 ΔNLS2-3HA-R2 | CACATCATAAGGATAGGTACCAAACATTTCATTACAATAAAAGTACTTCCCGTGC |
| ICP27 ΔKH1-3HA-F1 | CATCATTTTGGCAAAGAATTCGCCACCATGGCCTGCAGTGCTAAACCC |
| ICP27 ΔKH1-3HA-R1 | ATCGCATGACGCTAATGCAGCGGTTGTTCGGAAGCCTCCATT |
| ICP27 ΔKH1-3HA-F2 | AATGGAGGCTTCCGAACAACCGCTGCATTAGCGTCATGCGAT |
| ICP27 ΔKH1-3HA-R2 | CACATCATAAGGATAGGTACCAAACATTTCATTACAATAAAAGTACTTCCCGTGC |
| ICP27 ΔNES-3HA-F1 | CATCATTTTGGCAAAGAATTCGCCACCATGGCCTGCAGTGCTAAACCC |
| ICP27 ΔNES-3HA-R1 | AAAGCTCGGTAGGTCGACTATTATTAAGTCTTCATTGCGCAG |
| ICP27 ΔNES-3HA-F2 | CTGCGCAATGAAGACTTAATAATAGTCGACCTACCGAGCTTT |
| ICP27 ΔNES-3HA-R2 | CACATCATAAGGATAGGTACCAAACATTTCATTACAATAAAAGTACTTCCCGTGC |
| ICP27 ΔC-3HA-F | CATCATTTTGGCAAAGAATTCGCCACCATGGCCTGCAGTGCTAAACCC |
| ICP27 ΔC-3HA-R | CACATCATAAGGATAGGTACCATCGCCCTTGTCTCCTCTCTT |
| ICP27 ΔNC-3HA-F | CATCATTTTGGCAAAGAATTCGCCACCATGAAGAAAAAACCCTCCGATCAT |
| ICP27 ΔNC-3HA-R | CACATCATAAGGATAGGTACCATCGCCCTTGTCTCCTCTCTT |
| ICP27 mutZnF-3HA-F1 | CATCATTTTGGCAAAGAATTCGCCACCATGGCCTGCAGTGCTAAACCC |
| ICP27 mutZnF-3HA-R1 | CTCGAATACAATTTAGCCTGTTGGTCGTGAGCATCGTGCAAAGCTTCGTCTATCATCT |
| ICP27 mutZnF-3HA-F2 | ATGATAGACGAAGCTTTGCACGATGCTCACGACCAACAGGCTAAATTGTATTCGAG |
| ICP27 mutZnF-3HA-R2 | CACATCATAAGGATAGGTACCAAACATTTCATTACAATAAAAGTACTTCCCGTGC |
| Du EGFP-C2-RIG-I(N-Terminal)-F | GACTCAGATCTCGAGCTCAAGCTTAACGGCGGACGAGAAGCGGAGC |
| Du EGFP-C2-RIG-I(N-Terminal)-R | CAGTTATCTAGATCCGGTGGATCCTCACTCTTATATCCCACAGTTCACT |
| Du EGFP-C2-IRF7-F | GACTCAGATCTCGAGCTCAAGCTTAGCAGCGGCGGAGAGCGAAGGG |
| Du EGFP-C2-IRF7-R | CAGTTATCTAGATCCGGTGGATCCTCAGTCTATCTGCATGTT |
| Hu EGFP-C2-RIG-I(N-Terminal)-F | GACTCAGATCTCGAGCTCAAGCTTAACCACCGAGCAGCGACGCAGC |
| Hu EGFP-C2-RIG-I(N-Terminal)-R | CAGTTATCTAGATCCGGTGGATCCTCATTTCAAAGTTTTGGGCCAGTTT |
| Hu EGFP-C2-IRF3-F | GACTCAGATCTCGAGCTCAAGCTTAGGAACCCCAAAGCCACGGATC |
| Hu EGFP-C2-IRF3-R | CAGTTATCTAGATCCGGTGGATCCCTATTGGTTGAGGTGGTGGGGAAC |
| cGAS-F | CTACTACGAGCGCGTCAAGA |
| cGAS-R | CTGAATCCTCGCGATAGGCA |
| STING-F | GAGATGACCGAGAGGTCCCA |
| STING-R | ACACTCCTTTATGCGTGGCA |
| Du RIG-I-F | TCTCTGTCGGTCGGATAA |
| Du RIG-I-R | TCATCAGGTTCTGCTTCTTC |
| Hu RIG-I-F | CCAAAAAGCCACGGAACCAG |
| Hu RIG-I-R | GTGGGAGTTCGAGGTGACAG |
| MDA5-F | GCTGAAGAAGGCCTGGACAT |
| MDA5-R | TCCTCTGGACACGCTGAATG |
| MAVS-F | AGCCCAGAAATGAACCCCAG |
| MAVS-R | TCGAACTGCTGCTGGATGAG |
| TBK1-F | TTAGAGGAGCCATCCAACGC |
| TBK1-R | AGTTCTCTCGCAGCACCAAA |
| IRF7-F | AACGCCAGGAAGGATGTCAC |
| IRF7-R | CGCAGCGAAAGTTGGTCTTC |
| Hu IRF3-F | ACGTAGCTCATCACTCCCCT |
| Hu IRF3-R | AGGACAGGATGAACTTTGAC |
| IFN-β-F | TCTACAGAGCCTTGCCTGCAT |
| IFN-β-R | TGTCGGTGTCCAAAAGGATGT |
| OASL-F | TCTTCCTCAGCTGCTTCTCC |
| OASL-R | ACTTCGATGGACTCGCTGTT |
| Mx-F | TGCTGTCCTTCATGACTTCG |
| Mx-R | GCTTTGCTGAGCCGATTAAC |
| IL-6-F | TTCGACGAGGAGAAATGCTT |
| IL-6-R | CCTTATCGTCGTTGCCAGAT |
| UL55-F | GGTTGTTCTAGAGAGCGCGT |
| UL55-R | GCAAAGACGTTTCACGTCCC |
| ICP27-F | ACCTACAATTCAGCAACGCATA |
| ICP27-R | TGTTCGGAAGCCTCCATTCT |
| Lamin B1-F | AGGTTGATTCTGGTCGCCAA |
| Lamin B1-R | ATTCGCACGCGACTTTCATC |
| GAPDH-F | CACAGCCACACACGAAGACA |
| GAPDH-R | CCTTAGCCAGCCCCAGTAGA |
| 18S rRNA-F | GTACAGTGAAACTGCGAATGG |
| 18S rRNA-R | CGTCGGCATGTATTAGCTCTA |
